# Capacity and patient flow planning in post-term pregnancy outpatient clinics: a computer simulation modelling study

**DOI:** 10.1186/s12913-020-4943-y

**Published:** 2020-02-14

**Authors:** Joe Viana, Tone Breines Simonsen, Hildegunn E. Faraas, Nina Schmidt, Fredrik A. Dahl, Kari Flo

**Affiliations:** 1 Centre for Connected Care, Oslo University Hospital, Kirkeveien 166, 0450 Oslo, Norway; 2 Health Services Research Centre, Akershus University Hospital, 1478 Lørenskog, Norway; 3 Department of Obstetrics and Gynaecology, Akershus University Hospital, 1478 Lørenskog, Norway; 4 Institute of Clinical Medicine, Campus Ahus, University of Oslo, Lørenskog, Norway

**Keywords:** Simulation, Post-term pregnancies, Patient flow, Capacity planning, Optimisation, Outpatient

## Abstract

**Background:**

The demand for a large Norwegian hospital’s post-term pregnancy outpatient clinic has increased substantially over the last 10 years due to changes in the hospital’s catchment area and to clinical guidelines. Planning the clinic is further complicated due to the high did not attend rates as a result of women giving birth. The aim of this study is to determine the maximum number of women specified clinic configurations, combination of specified clinic resources, can feasibly serve within clinic opening times.

**Methods:**

A hybrid agent based discrete event simulation model of the clinic was used to evaluate alternative configurations to gain insight into clinic planning and to support decision making. Clinic configurations consisted of six factors: X0: Arrivals. X1: Arrival pattern. X2: Order of midwife and doctor consultations. X3: Number of midwives. X4: Number of doctors. X5: Number of cardiotocography (CTGs) machines. A full factorial experimental design of the six factors generated 608 configurations.

**Results:**

Each configuration was evaluated using the following measures: Y1: Arrivals. Y2: Time last woman checks out. Y3: Women’s length of stay (LoS). Y4: Clinic overrun time. Y5: Midwife waiting time (WT). Y6: Doctor WT. Y7: CTG connection WT. Optimisation was used to maximise X0 with respect to the 32 combinations of X1-X5. Configuration 0a, the base case Y1 = 7 women and Y3 = 102.97 [0.21] mins. Changing the arrival pattern (X1) and the order of the midwife and doctor consultations (X2) configuration 0d, where X3, X4, X5 = 0a, Y1 = 8 woman and Y3 86.06 [0.10] mins.

**Conclusions:**

The simulation model identified the availability of CTG machines as a bottleneck in the clinic, indicated by the WT for CTG connection effect on LoS. One additional CTG machine improved clinic performance to the same degree as an extra midwife and an extra doctor. The simulation model demonstrated significant reductions to LoS can be achieved without additional resources, by changing the clinic pathway and scheduling of appointments. A more general finding is that a simulation model can be used to identify bottlenecks, and efficient ways of restructuring an outpatient clinic.

## Background

### Context

The World Health Organisation (WHO) defines a pregnancy as post-term when the gestation period exceeds 42 (> 294 days) gestational weeks (GW) [1]. From 2008 to 2009 guidelines in the US, UK and in parts of Scandinavia were updated; women with low-risk pregnancies were offered induction of labour between GW 41 and 42 to avoid the risks of prolonged pregnancy [2–4]. The guidelines for post-term pregnancies in Norway were changed in 2011 [5]. The revised guidelines included an additional clinical examination in GW 41, 7–9 days after term, including ultrasonography and cardiotocography (CTG). Women > 38 years, women with a small for gestational age foetus (<below the 10% percentile), women with oligohydramnion, or pre-pregnant body mass index > 30 were offered induction immediately after the examination in GW 41. Generally, all inductions should be started before week 42 [5, 6]. If induction of labour is contraindicated due to medical reasons, the patient will be offered a caesarean section when appropriate due to the clinical context.

**Fig. 1:**
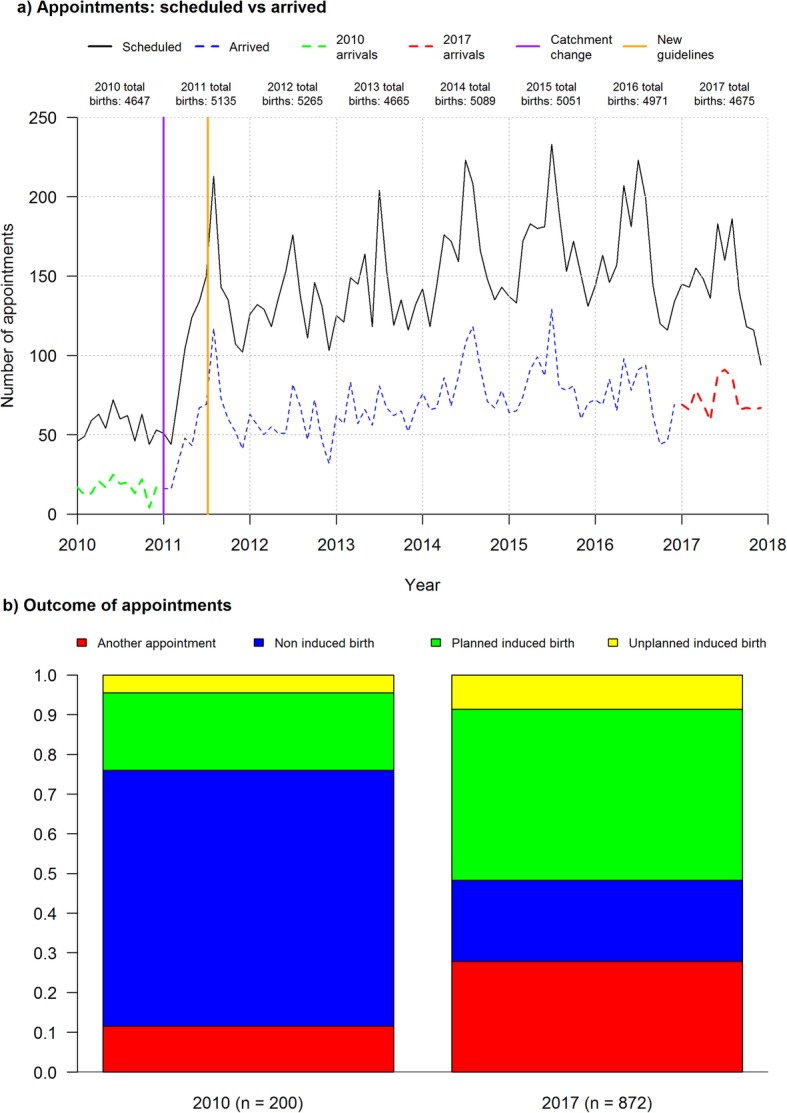
Changes in clinic demand and appointment outcomes from 2010 to 2017. Panel **a**) arrived vs. scheduled appointments, derived from hospital information systems DIPS and CSAM Partus based on ICDM-10-CM Dianosis Code O48.0.Panel **b**) appointment outcomes using subsequent O48.0 entries, and procedural codes for induced and non-induced births

The department of obstetrics and gynaecology at Akershus University Hospital (AHUS) manages women with post-term pregnancies at an outpatient clinic (the clinic) which is open weekdays between 08:00–10:30. The demand placed on the clinic has increased substantially since 2010. Figure 1a, illustrates over the period 2010–2017: the number of appointments scheduled and attended (arrived) as well as other key events including the change in the hospital’s catchment area and the introduction of the new post-term induction [5, 6] guidelines.

The outcome of clinic appointments (next hospital interaction) has changed over time, as illustrated in Fig. 1b, as the number of attended appointments increased by 436%, from 200 in 2010 to 872 in 2017. The outcomes of the appointments in 2010 were induced deliveries 24.00%, non-induced deliveries 64.50% and follow up clinic appointments 11.50%. The outcomes of the appointments in 2017 were 51.72, 20.41 and 27.86% respectively.

The new guidelines increased the number of appointments and the likelihood of appointment attendance. The gap between the scheduled appointment and attended appointment lines in Fig. 1a was reduced. The probability that women attended their appointment in 2010 (before the new guidelines) was 0.298 (200/671) and 0.472 (5787/12,266) in 2011–2017 (after guidelines). The difference in proportions is significant, χ^2^ (1, *N* = 18,924) = 77.235, *p* < 0.001. Attendance increased as appointments are scheduled earlier and more frequent, increasing the likelihood that women are able to attend.

Several studies have evaluated the policy of birth induction versus expectant management regarding perinatal outcomes, frequency of caesarean section, instrumental birth rate, etc. [7–12]. However, there is a lack of studies that investigated the operational impact on post-term pregnancy outpatient clinics after the change of guidelines.

In this computer simulation study, we developed a model of the post-term pregnancy clinic at AHUS, to evaluate clinic management strategies (patient flow). Simulation was chosen as it can capture the components of complex systems such as this one, which is subject to stochastic demand, arrival patterns and service times.

### Simulation modelling in health care

Computer simulation modelling approaches have been used in healthcare to provide virtual environments to evaluate changes in complex systems (a person, a ward within a department, a department, and a hospital) at operational, tactical and strategic management levels prior to considering changes in reality [13–15].

Each model is designed to answer a specific question(s) [16]. A series of modelling good research practice papers in healthcare have been written [17] and the discrete event simulation (DES) paper [18] is particularly relevant. DES is well suited for modelling operational level processes and is used in this paper. Jun et al. developed a tool to guide in the selection of the most appropriate Operational Research (OR) method(s), including simulation, to support health services management [19]. We follow the STRESS guidelines to document the model presented [20].

In the remainder of the background section we present selected obstetrics and gynaecology (OB/GYN) simulation models, split into three groups, training and economic evaluation models, inpatient delivery models and outpatient models, followed by a brief discussion of relevant outpatient models in other settings and a selection of the key scheduling literature.

### OB/GYN training and economic evaluation models

Simulation approaches have been used as a training aid to assist with the education of clinicians including, anatomical models which enable clinicians to practice procedures such as deliveries [21, 22], to demonstrate causal inference [23] and illustrated how experiential learning influences medical decision making [24]. Typically simulation and mathematical models are used in economic analyses of new and/or existing policies, including but not limited to, treatments, drugs or methods [17, 18]. Illustrative examples include the evaluation of screening strategies for the identification of foetal abnormalities [25], the effect of pooling units and changing capacities of an obstetric service [26] and planning the caseload at the individual midwife level [27].

### OB/GYN inpatient delivery models

A number of models of various levels of detail have been produced. The earliest DES of a maternity suite in a US hospital, focused on the impact of changes in demand, the type of demand and the availability of supply [28]. Another DES model examined specifically the optimal anaesthesia teams required in obstetrics, without detracting from the quality of care [29]. Other simulation approaches including Markov [30, 31] and mathematical modelling [32] have been used to examine patient flow in obstetrics departments and determine maternity length of stay (LoS) respectively.

Data mining and clustering analysis were proposed as a way to identify key groups, to be considered prior to simulation, mathematical or analytical analyses [33]. Several DES models of specific US obstetrics inpatient departments have been produced to support the delivery of more efficient services including, capacity planning, appointment scheduling, optimal bed allocation, use of flexible rooms, and scheduling of staff and operating rooms [34–37]. Other studies of note include a combined DES optimisation model of an Italian obstetrics ward to maximise the net profit, based on various of the caesarean and vaginal birth rates subject to certain constraints [38], an Australian DES focusing on the processes required to ensure that induction births take place with specified times [39] and the evaluation of a midwife staffing approach in the UK to achieve better flexibility with the existing staff capacity [40].

The models presented above are relevant to this paper as they are simulation models used in the same domain, but they focus on inpatient visits, which have different scheduling needs, and the anticipated LoS is measured in days, not minutes. They were all designed for a specific purpose to address specific question(s).

### OB/GYN outpatient models

The earliest model we identified, evaluated the planned merger of two clinics in the US [41]. This DES study found that the proposed merger was infeasible due to waiting and examination space requirements. Later a general framework for simulating obstetric outpatient clinics is proposed [42], however it is unclear if this framework has been adopted. The DES model developed to improve patient waiting time and flow in an OB/GYN outpatient clinic [43], could be improved by running more runs per scenario as DES to be more representative due to sampling from multiple distributions, and the number of “what-if” scenarios to evaluate could be expanded. Lenin et al. demonstrated through DES the optimised appointment templates for certain OB/GYN clinics [44]. The authors differentiated between three different patient types, each of which had different likelihoods of attending their appointments. The model was run over a week and the optimal number of staff and the time between appointments was assessed. The optimal solution required an additional Medical Assistant and the modification of the appointment system. This led to reduced waiting times by removing bottlenecks, without sacrificing the utilisation of resources. They were able to do this as they had access to high quality patient tracker data.

This paper extends an earlier paper by Viana et al. 2018 which introduced the post-term pregnancy outpatient clinic model that has been subsequently further developed [45]. This previous version of the model was validated against historical demand, before assessing the impact on certain performance measures (resource utilisation and time measures) with respect to three scenarios only, 1) increased demand, 2) an additional midwife and 3) a combination of 1 and 2. The scenarios were for illustrative purposes. This paper focuses on a vast number of alternative scenarios that are of greater interest to the department.

### Other relevant outpatient models

Two previously mentioned reviews reference several outpatient clinics [13, 14], these various outpatient settings are comparable with the post term pregnancy clinic in many ways although the no show/ did not attend (DNA) rates vary substantially. Although Mohiuddin et al. review simulation modelling of patient flow in emergency departments [15], these are somewhat comparable with outpatient models. Gunal and Pidd’s review of the use of DES in healthcare illustrates how the number of papers has increased significantly since the previous reviews [46]. What is clear from this review is that models developed for specific purposes dominate more generic approaches.

### Scheduling

Scheduling in health and social care settings is important to balance the trade of between patient waiting time and staff/resource idle time. Cayirli and Veral provide an overview of the use mathematical modelling, simulation and heuristic approaches to understand and improve outpatient scheduling [47]. Early seminal work on clinic appointment scheduling suggested that block scheduling where patients arrive at specified intervals, with a double booking in the first slot is preferable to all the patients arriving at the start of a clinic [48]. A weakness of some early scheduling work was the assumption of punctuality both in terms of the patients and the healthcare professionals; this was addressed in later works [49–52]. In addition to the issues caused by unpunctuality, the no show rate or DNA rate has a substantial effect on clinic performance [53]. Many of the studies introduced in the inpatient and outpatient sections include scheduling but the most relevant study to our context is the use of a DES model to optimise appointment scheduling in US OB/GYN clinic [44]. In this study the authors determined the optimal duration between appointments for set numbers of patients split into two sessions.

The configurations of the post term clinic in this study are assessed by a number of KPIs that shall be introduced later. The situation at present is that although women are given appointment times, staff have informed us that the majority of women who arrive, arrive when the clinic opens, negating the positive effects in terms of their waiting times and LoS.

## Methods

### Aim

The aim of this simulation study is to determine the maximum number of women specified clinic configurations, combination of specified clinic resources, can feasibly serve within clinic opening times.

The cost effectiveness of the configurations is outside the scope of this paper as we do not have access to patient outcome data. More nuanced appointment scheduling, to minimise the women’s waiting time and total LoS will be considered in future analysis. Due to the fluctuating DNA rate of women who miss appointments, as a result of giving birth, and the majority of women arrive before the clinic opens, the maximum number of women each configuration is considered the main model outcome.

### Study design and setting

A computer simulation modelling study was conducted.

The simulation model represents the operation of the post-term pregnancy outpatient clinic at AHUS, a large Norwegian hospital. Prior to the catchment area change in 2011 AHUS served ~ 300,000 people afterwards it served ~ 480,000 people, of which ~ 61,000 and ~ 96,000 were females aged 15–44 respectively [54–56].

### Characteristics of participants

The participants are hypothetical computer simulated pregnant women in GW 41, 7–9 days after term, who have been scheduled to attend the post-term pregnancy outpatient clinic.

### Model specification

The clinic operates between 08:00 and 10:30 four days a week. One day a week the clinic finishes at 09:30 as the doctors have to perform other tasks. The clinic operates on weekdays. In the current system appointments are given in 5 min intervals which begin at 08:00 and run up until 09:00, resulting in 12 possible appointments. However, as previously mentioned a challenge of operating this clinic is the high risk of DNAs due to women giving birth before appointments, and also the uncertain demand, so the range of appointments actual attended is 0–15. Additionally, although women are given appointment times, those that arrive on the day of their appointment tend to arrive when the clinic opens. The model is constructed to evaluate the maximum number of appointments that can be satisfied within clinic opening times on a typical day, 08:00 to 10:30. The scheduling and outpatient configuration literature discussed previously generally assumes higher attendance rates and less uncertain demand.

Following Norwegian guidelines a post-term pregnant woman, with a singleton pregnancy without complications, is given a set of appointments to attend the clinic for checks. Women with multiple pregnancies or known complications are monitored by alternative mechanisms. If the woman attends her appointment, the standard process is that she will check in at reception. She will be called in to the midwife consultation which consists of various tasks including a CTG scan once resources, rooms and equipment are available. Following the midwife consultation if the doctor is free the woman will be called into the doctor’s office, for the doctor consultation. At the end of the consultation, the doctor will either recommend the woman for an induced birth or a follow-up appointment a few days later. The woman will check out at reception, return home or be scheduled for an induction.

**Fig. 2:**
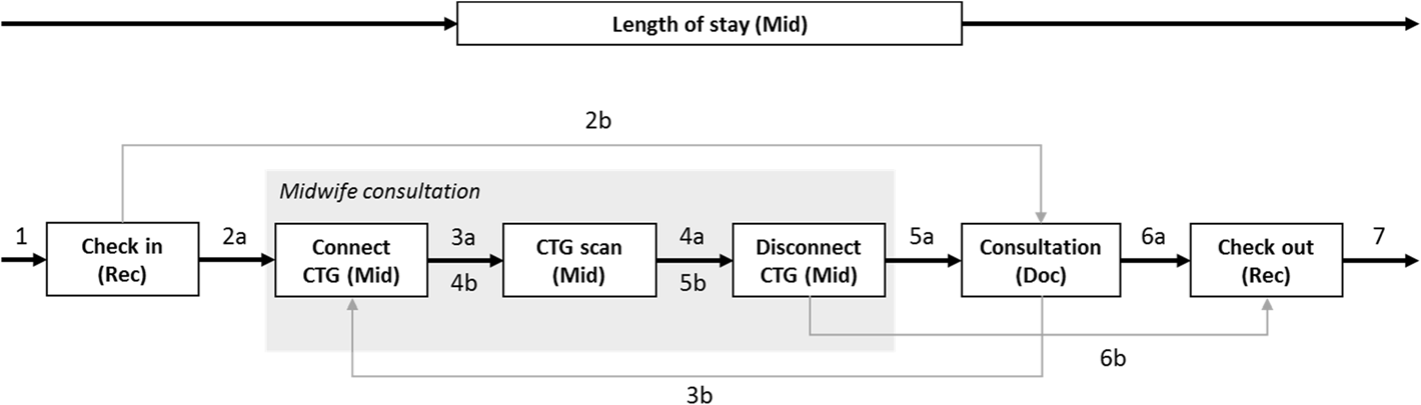
An illustration of the pathway through the overdue clinic. The numbers illustrate the sequence of activities. The current pathway is indicated with “a”. A proposed alternative pathway is indicated with “b”. The letters in brackets indicate which staff group delivers the activity, Doc = Doctors, Mid = Midwives, and Rec = Reception staff

An overview of the key components of the model is provided here. The hybrid Agent Based Discrete Event Simulation model is constructed in Any Logic Professional 8.3.3 [57]. DES is widely used to model systems in which entities in this case pregnant women pass through sequential activities that may be constrained by resources, e.g. space, staff, equipment. Due to resource constraints, variability in arrivals and activity service times, queues can form in these systems resulting in delays and bottlenecks. The clinic DES model captures the processes depicted in Fig. 2.

Each post-term pregnant woman in the model is represented as an agent from the Agent Based Modelling/Simulation (ABM/ABS) paradigm. A woman agent (WA) will be referred to as *woman* (or *women* where appropriate) in this paper to differentiate between computer simulated and actual women. In addition to post-term *women*, the model also simulates *women* belonging to other clinics, which for the present analysis only affect the flow of *women* by creating queues at check in and check out and competing for waiting resource, in the waiting area and corridors. Overall, the model is more detailed than indicated by Fig. 2, and includes e.g. a representation of the physical layout of the clinic, and even chairs in the waiting areas. For a more detailed model description see [45] and Additional file 1.

Each *woman* contains variables relating to its demographic and clinical characteristics, in additional to variable relating to its foetus. It was anticipated that these variables could be used to make more informed decisions, but data access and quality prevents the use of these variables in this paper. Each *woman* records information about its interaction with the DES clinic in relation to the activities depicted in Fig. 2, including the waiting time for and the duration of each activity. A *woman’s* length of stay (LoS) is the difference between arrival at and departure from the clinic which is equivalent to the sum of the *woman’s* recorded time intervals. The DES clinic model collects utilisation results for resources described in Fig. 2: reception staff, midwives, doctors, CTGs and waiting capacities. The model results collected at the *women* and the DES clinic model levels are collated at the end of each run of the simulation model and exported as csv files for further analysis.

#### Configuration

A model configuration relates to the six parts of the model that can be changed to assess clinic performance, with respect to a specific demand. The input values for each configuration are provided in the inputs section but this section introduces them briefly. A configuration consists of the type of arrival pattern the model uses to determine when the specified demand. As mentioned the clinic does use an appointment system but the women often arrive when the clinic opens. Two simplified appointment systems are considered in this paper. Changes to the pathway discussed in the model specification and are illustrated in Fig. 2. The pathways considered in this paper are those illustrated in Fig. 2, but alternative pathways could also be analysed with minimal effort. The other components of a configuration are the number of clinical staff, midwives and doctors and the number of CTG machines.

#### Data sources

To represent the clinic accurately in a simulation model, data relating to clinic processes and activities are required, specifically the pathways currently in operation, feasible alternative pathways, and probability distributions that represent the service times of the different processes in the model.

The electronic patient registry and the hospital information system do not collect time stamped data; we could use to derive duration times at the activity level required by the model for outpatient clinics. The hospital information system is geared towards the collection of inpatient data, the only time data we have for outpatient clinics are appointment times which are recorded consistently and as we have alluded to women tend to arrive when or before the clinic opens <=08:00.

We asked staff to provide their best estimates for the durations (service times) for various activities. They were in the best place to provide these as they serve this patient group daily and it was the only feasible way to get service time data for the model. Neither the clinic nor the research team have the resources at the time to conduct a time in motion study, whereby we would follow patients around the clinic recording their process times or devise a method to collect this information.

We therefore developed an Excel VBA program based on the method described by Leal et al. [58], to elicit estimates from employees. The tool was not used to record actual service times. In Fig. 2 the bracketed abbreviations indicate which estimates were provided by reception staff (Rec), midwives (Mid) and doctors (Doc). Five obstetricians, six midwives and five reception staff took part in the estimation exercise. Service time probability distributions created for: Check in, Connect CTG, CTG scan, Disconnect CTG, Consultation, and Check out, based on the input from the staff, these staff derived service time distributions were combined and validated through discussion with clinic management and the clinical authors of this paper. Descriptive statistics of the service time distributions used in the model and the number of CTG checks are provided in Table 1.

**Table 1:** Summary statistics of service time distributions (minutes) and number of CTG checks

Service	Min	Median	Mean	Max	Standard Deviation	Kurtosis	Skewness
Check In	0.130	0.467	0.457	1.000	0.249	2.156	0.531
Connect CTG	2.000	3.341	3.410	5.000	0.863	1.842	0.156
CTG Scan	20.000	25.557	26.250	45.000	4.855	6.011	1.558
Disconnect CTG	2.000	3.341	3.410	5.000	0.863	1.842	0.156
Consultation	10.000	21.282	22.325	45.000	4.901	4.764	1.005
Check out	0.130	1.580	1.589	5.000	0.985	2.696	0.480
No. of CTG checks	0.000	1.897	2.120	10.000	1.462	8.374	1.793

A more detailed description of how the service time distributions were derived is provided in Additional file 1. No waiting time estimates were elicited from clinic staff, as these are the results of queues in the system, and estimated by the simulation model.

#### Assumptions

The model assumptions listed below, are made to reduce the scope of the system to its key factors.

- The clinic’s midwives and doctors did not attend to other non-clinic *women* during clinic hours.
- The reception staff and waiting area resources (waiting area chairs and corridor space) are shared with *women* attending all clinics.
- The midwives are able to attend to multiple *women*.
- A midwife will stay with a woman who requires 5 or more CTG checks, (mean number checks is 2.120). A summary of the CTG distribution is provided in Table 1 and the distribution in the Additional file 1.
- If a *woman* sees a doctor before a midwife, the *woman* does not have to see a doctor again after the midwife consultation. It was assumed that the doctor has sufficient information to complete the consultation.
- The clinic will stay open if *women* still need to use it. Results are collected within and outside clinic hours. In reality staff would have other duties to attend to. The simulation model runs until midnight to treat as many *women* as possible. Configurations that fail to satisfy the demand in clinic hours are rejected for that level of demand.
- All clinic appointments are attended. This places greater stress on each configuration, as in reality women may miss their appointment as they have given birth prior to it. Therefore all of the results presented are based on the maximum number of women each configuration can serve within clinic opening hours.
- The model does not differentiate between new and returning patients. New patients are those referred to the clinic for their first appointment for the current pregnancy. Returning patients are those attending their second, third etc. appointment for current pregnancy. The service time estimates provided by staff are for all clinic appointments.
- Two simplistic appointment systems are assessed in this paper. That either all of the demand arrives when the clinic opens at 08:00 or half of the demand arrives at 08:00 and half at 08:30. More nuanced appointment systems can be evaluated, but in general if the women arrive they tend to arrive before the clinic opens.
- The queuing discipline currently employed in the model is first in first out (FIFO).
- The model of the clinic operates a single queue multiple server system. Women are not assigned to particular midwives, CTG machines or doctors.
- The model represents the clinic in isolation, and does not take into consideration the occupancy of the maternity ward or operating theatres with respect to induction decision. This is outside the scope of this model.

#### Validation

Since detailed time stamp data is not collected for the clinic, we could not validate the model against historical data. As a substitute for this, we validated the model through visual demonstrations of the model during the construction process, and the clinic staff concluded that the model had a high degree of face validity. We performed extreme value tests, including: 1) very large/small arrival numbers at the clinic, checking queues formed in the model where appropriate, 2) excessive/insufficient amounts of resources (e.g. doctors etc.) and checked if the model outcomes were as expected. Additionally, we varied the activity services distributions, between (un)favourable alternatives and verified this had the expected effects on a *woman’s* visit. Finally, we validated the *women’s* average LoS against the LoS estimate provided by the midwives. For the baseline configuration (configuration 0a, Table 1) the model output matched the estimates well. This suggests that the sum of the waiting times (which are based on the simulated queues in the system, rather than pre-specified times) match the real waiting times well.

### Experimental design

#### Time horizon

The model can run for any time horizon. In this paper it runs, in minutes, for a single clinic day, from 08:00 to 23:59. Utilisation results for resources (e.g. midwives etc.) are recorded during clinic hours 08:00 to 10:30. It runs until 23:59 to allow all *women* who attend the clinic to be seen, the clinic may not be able to satisfy the demand if it is poorly configured or if demand is too great. The simulation starts with an empty clinic, which reflects the reality, since there are no overnight stays. A warm-up period for the model was also not considered necessary.

#### Multiple runs

Due to the randomness in a single model run, we collected statistics from 10,000 runs (model trial) for each model configuration. Randomness in this model relates to the service times. It is necessary to run the model multiple times for each configuration to avoid basing decisions on a potentially unrepresentative single run of the model.

#### Inputs

Through discussion with clinic staff, six inputs (decision variables) were defined. A clinic configuration is the values these inputs take. The inputs and the values they can take are described in Table 2.

**Table 2:** Model inputs

Input	Description	Values
X0	Arrivals. The number of *women* who attend the clinic.	- No default value, it ranges from 2 to 20. Also a Key Performance Indicator (KPI), see next section.
X1	Arrivals pattern	- **“All”**^a^. All the *women* arrive at 08:00 - “Half”. Half of the *women* arrive at 08:00 and half at 08:30 For odd numbers of arrivals the larger value arrives at 08:00.
X2	Order of midwife and doctor consultation	- “Doc” a *woman* can see a doctor first if one is available, before seeing a midwife - **“Mid”**^a^ the *woman* sees a midwife before a doctor.
X3	Number of midwives	**Two**^a^ or three.
X4	Number of doctors	**Two**^a^ or three.
X5	Number of CTGs	**Three**^a^ or four.

^a^Base case indicated in bold

A full factorial experimental design for the inputs described above results in 608 configurations. Each of these configurations was evaluated by a model trial.

### Key performance indicators

The configurations are evaluated using seven model KPIs. The average KPIs derived from a model trial per configuration are stated in Table 3. Waiting time KPIs are collected to indicate where potential bottlenecks may exist.

**Table 3:** Model KPIs – Average results from 10,000 runs of the model

KPI	Description
Y1	Arrivals. Number of women attending clinic (same as X0, see *Optimal Treatment Capacity section*)
Y2^a^	Time last *woman* checks out (HH:MM:SS)
Y3	Women’s LoS (mins)
Y4^b^	Clinic overrun (mins after 10:30)
Y5	Women’s midwife waiting time (mins) [CTG connection wait + CTG disconnection wait].
Y6	Women’s doctor waiting time (mins)
Y7	Women’s CTG connection waiting time (mins) [CTG wait + midwife wait for connection]

^a^ Y2 explanation: Consider a scenario where 7 *women* arrive (10,000 model runs (a model trial) simulates 70,000 *women*). Say for 5000 runs the time the last *woman* leaves is 10:35 and for 5000 runs the time the last *woman* leaves is 10:20. The average of the model trial would be 10:27:30

^b^ Y4 explanation: In the 5000 runs where the last *woman* leaves at 10:35 at least one of the *women* left late e.g. after 10:30. Assuming that only ONE *woman* in each of these runs finished 5 min late, results in an average clinic overrun time of 0.5 min (7000 *women* [1 *woman* leaving late each run] * 5 min) / 70,000 *women* [the total number of *women*] = 0.5 min)

### Optimal treatment capacity

The six model decision variables (X0, X1, X2, X3, X4, and X5) described generated 608 configurations. The arrivals input (X0) ranges from 2 to 20 *women* (19 arrival patterns). Optimisation was performed on the configurations where X1, X2, X3, X4 and X5 were set and X0 incremented. This results in 32 configurations (608/19). This is illustrated in Table 4.

**Table 4:** Optimal treatment capacity

Inputs	Input levels	Cumulative configurations
X1: Arrival pattern	2	2
X2: Order of midwife and doctor consultations	2	4
X3: Number of midwives	2	8
X4: Number of doctors	2	16
X5: Number of GTGs	2	32^a^
X0: Arrivals	19	608^b^

^a^ results presented in this paper

^b^ results available in Additional file 2

The goal of the optimisation was to maximise the number of *women* (X0/Y1) that could be seen subject to the average time the last *woman* checks out (Y2) ≤10:30 and minimising the average clinic overrun time (Y4). This provided the optimal number of *women* each of the 32 configurations could serve. The ability to vary the model demand enables the effects of increases that may arise due to changes in the catchment areas or changes to guidelines to be evaluated.

The simulation model output is recorded in a number or text files for each model trial for each configuration. This large number of files is then analysed post hoc using R to produce outputs and enable the team to examine individual patient responses in a particular run in a particular trial if necessary. More information about how AnyLogic interacts with R is provided in Additional file 1.

## Results

The 32 configurations have been divided into eight groups (0–7) based on the number of midwives (X3), doctors (X4) and CTG machines (X5). The groups are shown in Table 5.

**Table 5:** Groups of configurations

Group	Purpose	Midwives (X3)	Doctors (X4)	CTGs (X5)
0	The base case. The number of resources currently available.	2	2	3
1	Evaluates the effect of one extra midwife.	3	2	3
2	Evaluates the effect of one extra doctor.	2	3	3
3	Evaluates the effect of one extra midwife and doctor.	3	3	3
4	Evaluates the effect of one extra CTG machine.	2	2	4
5	Evaluates the effect of one extra midwife and one extra CTG machine.	3	2	4
6	Evaluates the effect of one extra doctor and one extra CTG machine.	2	4	4
7	Evaluates the effect of one extra midwife.	3	3	4

Each group is divided into four subgroups (a-d) based on the combination of the X1 (Arrivals at 08:00) and X2 (Midwife or doctor first) inputs. The subgroups are described in Table 6.

**Table 6:** Group subgroups

Subgroup	Description	X1	X2
a	The base case. All *women* arrive at 08:00 and they all see the midwife before seeing the doctor.	“All”	“Mid”
b	Half the *women* arrive at 08:00 and half at 08:30 and they all see the midwife before seeing the doctor.	“Half”	“Mid”
c	All the *women* arrive at 08:00 and they can see a doctor first if a doctor is free.	“All”	“Doc”
d	Half the *women* arrive at 08:00 and half at 08:30 and they can see a doctor first if a doctor is free.	“Half”	“Doc”

Table 6 and Table 7 provide configuration inputs and the averages of the models KPIs based model trials with confidence intervals (95% level) for the 32 configurations. The base case (configuration 0a) is highlighted in bold in the respective tables.

**Table 7:** Mean time KPIs (from model trials) from selected configurations (base case in bold)

	Inputs						Key performance indicators					
Configuration	X1 – Arrivals at 8:00	X2 – Midwife or doctor first	X3 – Midwives	X4 – Doctors	X5 – CTGs	X0/Y1 – Arrivals / Treatment capacity (*women*)	Y2 Time last *woman* checks out (CI in seconds)		Y3 *Women’s* LoS (minutes)		Y4 Clinic overrun (minutes)	
							Mean	±	Mean	±	Mean	±
0a	All	Mid	2	2	3	7	10:25:33	11	102.97	0.21	2.00	0.09
0b	Half	Mid	2	2	3	7	10:25:36	10	90.13	0.14	2.03	0.09
0c	All	Doc	2	2	3	8	10:18:18	12	98.66	0.15	0.81	0.06
0d	Half	Doc	2	2	3	8	10:22:29	11	86.06	0.10	1.27	0.07
1a	All	Mid	3	2	3	7	10:21:17	10	99.72	0.20	0.76	0.05
1b	Half	Mid	3	2	3	7	10:21:26	10	86.99	0.13	0.78	0.05
1c	All	Doc	3	2	3	9	10:27:00	11	101.47	0.16	2.49	0.09
1d	Half	Doc	3	2	3	9	10:28:58	10	91.41	0.09	3.18	0.11
2a	All	Mid	2	3	3	7	10:23:44	11	99.90	0.20	1.67	0.08
2b	Half	Mid	2	3	3	7	10:23:46	12	87.15	0.14	1.67	0.08
2c	All	Doc	2	3	3	9	10:22:13	13	100.08	0.16	1.84	0.09
2d	Half	Doc	2	3	3	9	10:27:44	12	88.64	0.11	3.22	0.12
3a	All	Mid	3	3	3	8	10:27:30	10	101.59	0.21	2.37	0.09
3b	Half	Mid	3	3	3	8	10:29:13	10	87.72	0.14	3.04	0.10
3c	All	Doc	3	3	3	9	10:12:28	10	95.33	0.14	0.19	0.03
3d	Half	Doc	3	3	3	9	10:23:38	11	86.15	0.10	1.34	0.07
4a	All	Mid	2	2	4	8	10:28:21	12	105.93	0.20	3.31	0.12
4b	Half	Mid	2	2	4	8	10:28:33	12	91.09	0.13	3.47	0.12
4c	All	Doc	2	2	4	9	10:16:35	14	99.86	0.13	1.10	0.07
4d	Half	Doc	2	2	4	9	10:18:38	13	88.64	0.09	1.24	0.08
5a	All	Mid	3	2	4	8	10:22:16	10	101.57	0.19	0.99	0.06
5b	Half	Mid	3	2	4	8	10:22:23	10	86.71	0.11	1.03	0.06
5c	All	Doc	3	2	4	10	10:24:36	12	100.28	0.14	1.94	0.09
5d	Half	Doc	3	2	4	10	10:26:10	11	88.27	0.10	1.99	0.09
6a	All	Mid	2	3	4	8	10:17:27	14	99.49	0.18	1.34	0.08
6b	Half	Mid	2	3	4	8	10:17:25	14	84.57	0.11	1.30	0.08
6c	All	Doc	2	3	4	11	10:28:38	14	104.54	0.15	4.24	0.16
6d	Half	Doc	2	3	4	10	10:21:54	14	86.55	0.10	2.18	0.11
7a	All	Mid	3	3	4	10	10:29:03	10	103.05	0.18	2.83	0.10
7b	Half	Mid	3	3	4	10	10:29:05	9	88.04	0.12	2.84	0.10
7c	All	Doc	3	3	4	12	10:27:43	10	101.30	0.14	2.39	0.09
7d	Half	Doc	3	3	4	11	10:21:57	10	85.47	0.08	1.00	0.06

The following mean time related KPIs are provided in Table 7: time last *woman* checks out (Y2), *Women’s* LoS (Y3) and clinic overrun (Y4), in addition to the main output, arrivals/treatment capacity (X0/Y1). The base scenario has the highest LoS given its optimal treatment capacity. Generally, the “d” configurations in each group had a higher treatment capacity and a lower LoS compared to the “a” configurations. Configuration 0d which has the same resources as the base case 0a, can see an additional *woman* and reduces the mean LoS by 16.91 min (86.06 [0.10] vs. 102.97 [0.21] minutes).

The following mean waiting time related KPIs are provided in Table 8: *Women’s* midwife waiting time (Y5), *Women’s* doctor waiting time (Y6) and *Women’s* CTG connection waiting time (Y7), in addition to the main output, arrivals/treatment capacity (X0/Y1). The base scenario has the highest CTG connection waiting time given its treatment capacity. Generally, the “d” configurations in each group had lower cumulative waiting times (Y5 + Y6 + Y7) compared to the “a” configurations.

**Table 8:** Mean waiting time KPIs (from model trials) from selected configurations (base case in bold)

	Inputs						Key performance indicators					
Configuration	X1 – Arrivals at 8:00	X2 – Midwife or doctor first	X3 – Midwives	X4 – Doctors	X5 – CTGs	X0/Y1 – Arrivals / Treatment capacity (*women*)	Y5 *Women’s* midwife waiting time (minutes)		Y6 *Women’s* doctor waiting time (minutes)		Y7 *Women’s* CTG connection waiting time (minutes)	
							Mean	±	Mean	±	Mean	±
0a	All	Mid	2	2	3	7	2.54	0.04	3.11	0.04	29.48	0.19
0b	Half	Mid	2	2	3	7	2.54	0.04	3.09	0.04	17.23	0.12
0c	All	Doc	2	2	3	8	2.42	0.03	14.69	0.12	12.96	0.10
0d	Half	Doc	2	2	3	8	1.24	0.02	7.88	0.09	7.94	0.07
1a	All	Mid	3	2	3	7	0.06	0.00	4.31	0.05	25.62	0.18
1b	Half	Mid	3	2	3	7	0.06	0.00	4.34	0.05	13.38	0.12
1c	All	Doc	3	2	3	9	0.06	0.00	16.60	0.13	14.32	0.13
1d	Half	Doc	3	2	3	9	0.07	0.00	10.39	0.09	11.19	0.09
2a	All	Mid	2	3	3	7	2.53	0.04	0.01	0.00	29.47	0.19
2b	Half	Mid	2	3	3	7	2.55	0.04	0.01	0.00	17.26	0.12
2c	All	Doc	2	3	3	9	2.50	0.03	8.35	0.06	20.34	0.11
2d	Half	Doc	2	3	3	9	1.42	0.02	5.22	0.06	12.74	0.08
3a	All	Mid	3	3	3	8	0.07	0.00	0.01	0.00	31.65	0.19
3b	Half	Mid	3	3	3	8	0.09	0.00	0.01	0.00	17.85	0.13
3c	All	Doc	3	3	3	9	0.07	0.00	9.05	0.06	15.51	0.09
3d	Half	Doc	3	3	3	9	0.03	0.00	5.29	0.06	10.80	0.07
4a	All	Mid	2	2	4	8	6.22	0.06	7.67	0.06	26.90	0.17
4b	Half	Mid	2	2	4	8	6.24	0.06	7.70	0.06	12.63	0.09
4c	All	Doc	2	2	4	9	5.95	0.05	12.66	0.08	15.31	0.11
4d	Half	Doc	2	2	4	9	4.46	0.05	7.03	0.07	10.36	0.08
5a	All	Mid	3	2	4	8	1.46	0.02	10.78	0.07	20.60	0.14
5b	Half	Mid	3	2	4	8	1.46	0.02	10.80	0.07	6.27	0.05
5c	All	Doc	3	2	4	10	1.31	0.02	12.63	0.08	16.65	0.13
5d	Half	Doc	3	2	4	10	0.46	0.01	9.40	0.07	8.80	0.08
6a	All	Mid	2	3	4	8	6.26	0.06	1.18	0.02	27.00	0.17
6b	Half	Mid	2	3	4	8	6.21	0.06	1.17	0.02	12.61	0.09
6c	All	Doc	2	3	4	11	6.01	0.05	12.58	0.09	19.32	0.09
6d	Half	Doc	2	3	4	10	3.95	0.04	4.92	0.05	9.99	0.07
7a	All	Mid	3	3	4	10	1.30	0.02	1.59	0.02	30.62	0.17
7b	Half	Mid	3	3	4	10	1.32	0.02	1.60	0.02	16.52	0.10
7c	All	Doc	3	3	4	12	1.19	0.02	17.44	0.12	12.04	0.08
7d	Half	Doc	3	3	4	11	0.46	0.01	7.33	0.07	7.78	0.05

## Discussion

### Main findings

We have presented a simulation model developed to determine the capacity of and evaluate changes in the flow of post-term women through the clinic given alternative feasible configurations suggested by the clinic. We found that it is possible to improve the clinic’s performance with relatively small changes and at a low cost by changing pathways (X2 = “Mid”: patients see the midwife first; X2 = “Doc”: patient can see a doctor before a midwife if a doctor is available) rather than increasing the resources.

The simulation model was developed to inform decision-making not make the decisions for the clinic management. Many of the configurations presented in Table 1 and Table 2 are better than the base configuration (0a). It should be highlighted that configurations 0b, 0c and 0d which have the same number of midwives (X3), doctors (X4) and CTG machines (X5) as the base case (0a) perform better with respect to *women’s* LoS. Configurations 0c and 0d where *women* can see a doctor first before a midwife if a doctor is free (X2 = “Doc”) enables the clinic to see an additional woman within clinic opening times. For those configurations (0b, 1a, 1b, 2a and 2b) where the treatment capacity (Y1 = seven women) is the same as the base case (0a) the majority of the KPIs (Y2-Y7) are better. Unsurprisingly increasing clinic resources, midwives, doctors and CTG improves performance with respect to some KPIs.

We will highlight configuration 0d. This configuration does not involve additional resources, but changes to the arrival pattern (X1) of WAs and their pathway through the clinic (X2). This configuration is superior to the base configuration in many ways, an additional *woman* can be seen (0a = 7, 0d = 8), LoS is reduced by 16.4% (0a = 102.97 mins, 0d = 86.06 mins), average clinic overrun is reduced by 36.6% (0a = 2.00 mins, 0d = 1.27 mins) and the waiting time for the midwife and CTG connection is reduced by 51.12% (0a = 2.54 mins, 0d = 1.24 mins) and 73.06% (0a = 29.48 mins, 0d = 7.94 mins) respectively. The reductions in waiting times can be attributed to the changes in pathway (X2). Due to the additional *woman* who can be seen in this configuration the waiting time for the doctors increases by 153.5% (0a = 3.11 mins, 0d = 7.88 mins), and the utilisation of the doctors increased.

It is important to consider the KPIs as a whole, e.g. configuration 6b produced the best LoS (84.57 mins), with an additional doctor and CTG machine, but has a relatively high midwife waiting time as a result. It does enable the clinic to see eight *women* compared to seven, but there are other configurations that have similar LoS and higher treatment capacity, configurations 5d, 6d, 7b and 7d can see 10 *women*.

The model results provide valuable information for the clinic management. Additionally, the process of constructing the model with clinic staff highlighted the importance of considering uncertainty when planning how the clinic operates.

### Strengths and limitations

We actively involved clinic staff in the model development, from the development of the data estimation tool, to derive service time probability distributions, through the validation of the model. The clinic staffs engagement has increased the likelihood of the model results being used to support decision making, and also return to the model to evaluate other scenarios in the future. The developed clinic model considers the complexity in the system, which is more challenging to represent in analytical and statistical approaches.

The combination of a full factorial experimental design and the optimisation method were beneficial in terms of distilling large amounts of information into a more presentable form. The model was automated in such a way that you can run a batch of experiments of interest, by providing a set of input parameters to the model. The analysis of the simulation output was partially automated so it can be summarised succinctly in the statistical software package R. This post-hoc analysis outside the simulation environment is beneficial in terms of the variety of ways information from the model can be presented to stakeholders. It also frees up computational resources, so additional experiments can be conducted if necessary. A drawback of this approach how it is currently implement is that the optimisation takes place in R; after all of the model trials have been conducted in AnyLogic.

The model runs until midnight to ensure that all the women who arrive at the clinic are treated. This rather arbitrary decision does not affect run time as it is a DES which is an event based system so after the last woman is treated it advances to the end of the day. The latest time a *woman* left the clinic was 13:19:17, in configuration 0b when 20 *women* arrive. A computationally more efficient model could be developed.

The model presented included four inputs (X0, X3, X4, and X5) that were quantitative e.g. changing the number of resources available or the number of women who arrive, it also presented two inputs that were more qualitative in terms of how the clinic/system is configured (X1 and X2). The model can combine multiple inputs at different levels in addition to those presented in this paper and assess the effect these have on clinic performance. For example the capacity in the delivery and operating rooms, changes to service time distributions to reflect different patient types, i.e. new or returning, level of Norwegian etc.

The availability and the quality of data was a major challenge. The majority of the model data, e.g. service time and LoS distributions were elicited through estimation exercises with clinic staff. On one hand this was very beneficial as it engaged staff in the model building process. On the other hand the quantity and quality of outpatient data could be improved to be on a similar level as inpatient data, e.g. time stamps to record a patient’s journey through an outpatient clinic that we could then derive service time distributions from. It would be interesting to compare the service time estimates used in the model with actual service times. We would recommend that routine data is collected for all outpatient clinics not just the post-term pregnancy clinic, to enable quality of improvements of outpatient clinics. This is particularly pressing as healthcare shifts from an inpatient to an outpatient mode of delivery.

### Interpretation and further work

The model was constructed in collaboration with the clinic to help understand how it is performing. The process of building the model was challenging due to the availability of data. The model was developed to address particular questions posed by the AHUS post-term pregnancy clinic, it is bespoke. Although the model results have been presented and discussed with clinic management at the time of writing our recommendations have not yet been implemented.

The 608 clinic configurations evaluated were of greater interest to the clinic than the 4 scenarios described in earlier work [45]. As previously indicated the clinics notional appointment system splits the 08:00 to 09:00 period into 5 min intervals resulting in a maximum 12 appointments if single block booking was assumed. The base configuration has been shown in the model to serve a maximum of 7 women within clinic opening times. It should be noted that firstly it is extremely rare that 12 appointments are scheduled and secondly if 12 appointments were schedule is would be highly unlikely that all 12 would attend, due to the high DNA rate associated with this clinic. The results for the base case (configuration 0a) with 12 *women* arriving are as follows: Treatment Capacity (Y1) is 12, Time last woman checks out (Y2) is 11:30:48 [13.13 s], Women’s LoS (Y3) is 136.41 [0.26], Clinic overrun (mins after 10:30) (Y4) is 60.81 [0.21], Women’s midwife waiting time (mins) (Y5) is 2.38 [0.03], Women’s doctor waiting time (mins) (Y6) is 3.52 [0.03] and Women’s CTG connection time (mins) (Y7) is 61.42 [0.25].

In the model we assumed that the women scheduled to arrive at the clinic always did. We also assumed that in half of the 608 configurations based on observation of the clinic and feedback from staff most women arrived before the clinic opened (X1 = “All”). Assuming all patients arrived is the best case scenario in terms of numbers of patients treated. It is also represents the maximum stress a configuration of the clinic could efficiently serve, within clinic opening times. In reality women may miss their appointments as they have given birth. It would be useful as a theoretical exercise to examine alternative scheduling algorithms, now that we know how many women can be seen given a clinic configuration, to investigate if we can reduce waiting time and LoS.

The staff actively engaged in the development and validation of the model, the model at present needs to be run by the modeller. Upon further validation of the model a JAVA based web version of the model could be developed to allow staff at the clinic to experiment with different clinic configurations, using familiar office like interfaces. The model will need to be continually reassessed to ensure that it still accurately represents the operation of the clinic, and also to update it with more accurate data as and when it is available.

The model does provide many useful KPIs, but economic outputs are lacking. It would be beneficial to conduct a full cost effectiveness analysis, but at this time we do not have access to patient outcome data or cost data. If such information is available it can be added to the model. Currently equipment appears more of a bottleneck than personnel, as one additional CTG machine (group 4) has the same effect as an extra midwife and an extra doctor (group 3), and the waiting time for CTG connection is a key contributor to LoS.

The additional model variables associated with each *woman* e.g. demographic parameters, clinical measures/observations of the *foetus* can be used to identify different patient groups and the impact they have on clinic performance.

The increase in the number of appointments does not only affect the delivery of care at the clinic. Clinic decision makers rightly have to take into account the available resources of the maternity ward, such as delivery rooms, surgical and recovery rooms, and medical staff when they schedule induced births. It would be interesting to model the induction decision rules and pathways in a larger scale simulation model in the future. It may also be beneficial to look deeper into statistical and cost benefit analyses of the increase in demand due to the changes in catchment area and change of clinical guidelines.

## Conclusions

From an AHUS perspective, the changes in catchment area and guidelines for post-term pregnant women led to an increased work load for the clinic. The simulation model demonstrated that flexible pathways in the order of midwife/doctor and appointment scheduling increases flow substantially. Equipment appears more of a bottleneck than personnel, as one additional CTG machine has the same effect as an extra midwife and an extra doctor, and the waiting time for CTG connection is a key contributor to LoS. We are in discussion with clinic management about further developing the model, incorporating better quality data, evaluating alternative scenarios and implemented promising model suggestions. A more general finding is that a simple simulation model can be used to identify bottlenecks, and identify efficient ways of restructuring an outpatient clinic.

## Supplementary information

**Additional file 1.** Post-term pregnancy clinic model description; Provides a more detailed description of the model, the data and the experimental design.

**Additional file 2.** Summary model results; Provides mean results and errors for each KPI, by experiment.

**Additional file 3.** Mendeley Data; Provides a link to a Mendeley repository where the model, the results and the documentation used in this paper can be found.

**Additional file 4.** Explanation of Fig. 1 data; Provides an explanation of how Fig. 1 was constructed and summary data.

## Data Availability

The model and datasets generated and/or analysed during the current study are available in the Mendeley repository, https://data.mendeley.com/datasets/4jc5hz4mp3/1 [59]., see Additional file 3. The computer simulation model inputs are provided in Additional file 1. The datasets used to generate Fig. 1 analysed during the current study are not publicly available due to confidentiality and privacy regulations. Summary information about Fig. 1 is provided in Additional file 4.

## Abbreviations

ABM: Agent Based Model(ling)
ABS: Agent Based Simulation
CSAM: Clinical Systems All Managed. A Norwegian company specialising in Electronic Health Record System. CSAM Partus is a maternity information system
csv: Comma separated values
CTG: Cardiotocography
DES: Discrete event simulation
DIPS: Distribuert Informasjons og Pasientdatasystem i Sykehus (Distributed Information and Patient data system in Hospitals). A Norwegian company specialising in Electronic Health Record System
DNA: Did not attend
Doc: Doctor(s)
GW: Gestational week(s)
ICDM-10-CM: International Classification of Diseases, Tenth Revision, Clinical Modification
KPI: Key performance indicator(s)
LoS: Length of stay
Mid: Midwife/Midwives
mins: Minutes
O48.0: ICD-10-CM Diagnosis Code for Post-term pregnancy
Rec: Reception staff
UK: United Kingdom
US: United States of America
VBA: Visual Basic for Applications
WA: Woman agent
WHO: World Health Organisation
χ^2^: Chi square statistical test
