# Supplementary material for: Capacity and patient flow planning in post-term pregnancy outpatient clinics: a computer simulation modelling study
Source: BMC Health Serv Res. 2020 Feb 14;20:117. doi: 10.1186/s12913-020-4943-y (PMC7023739; doi:10.1186/s12913-020-4943-y)
Supplement: Supplementary file 1 — Additional file 1. Post-term pregnancy clinic model description; Provides a more detailed description of the model, the data and the experimental design. [file 12913_2020_4943_MOESM1_ESM.docx]

**Additional file 1 – Post-term pregnancy clinic model description**

Contents

[Introduction 1](#_Toc2724803)

[Data: Service time distributions estimation tool 2](#_Toc2724804)

[Data: Service time distributions 3](#_Toc2724805)

[Model: Class Structure 7](#_Toc2724806)

[Model: Graphical overview 8](#_Toc2724807)

[Full Factorial experimental design 9](#_Toc2724808)

# Introduction

This appendix contains more detail about the model, in particular the elicitation of the process time distributions from clinic staff. It is not possible to provide complete information to allow the model to be reproduced, but the model and associated code can be made available upon request. Unfortunately you will need AnyLogic Professional version 8.3.3 to run the model.

# Data: Service time distributions estimation tool

A programme was developed in Microsoft Excel using VBA to elicit estimates from clinic staff as data was not readily available. A screenshot from the programme is provided in Figure 1.


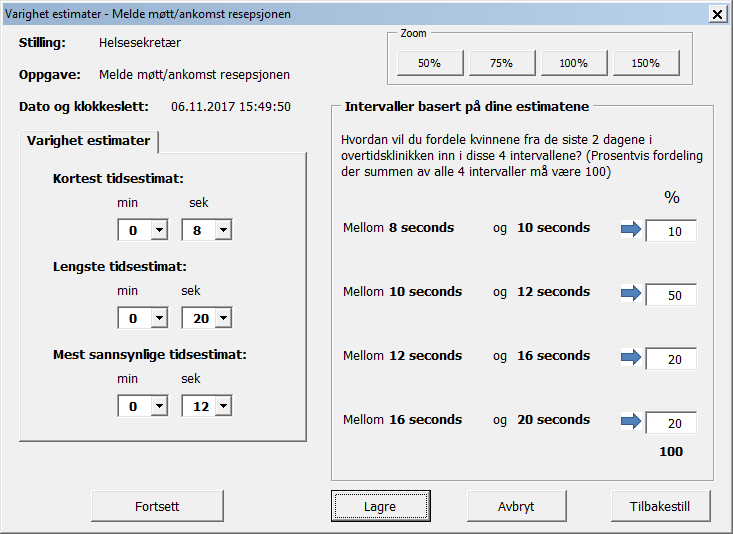


Figure 1 Screenshot from process time estimation program (only available in Norwegian)

Using the program process time distributions were derived for the processes depicted in Figure 2 of the paper and in the *Model: Graphical overview* section. The estimates used are shown in the *Data: Service time distributions* section. Table 1 below provides English translations of the Norwegian graph titles presented in this report.

**Table 1** Translation of graph titles

| Norwegian | English |
| --- | --- |
| Melde møtt/ankomst resepsjonen | Check in |
| Sjekk ved CTG_tidsbruk | CTG duration |
| Koble fra CTG | CTG disconnection |
| Tidsbruk legekonsultasjon | Doctor consultation |
| Avslutte time | Check out |
| Total tidsbruk overtidskontroll | Length of stay |
| Sjekk ved CTG_antall | CTG number of checks |

The service time distributions depicted in Figures 2, 4, 8 and 9 were provided through the data elicitation program. The decimal delimiter in the associated tables is a “,”. The service distributions depicted in Figure 3, 5, 6 and 7 were provided by TBS and KF authors of this paper. The x axes in the graphs are in minutes apart from Figure 4 which is the frequency.

# Data: Service time distributions


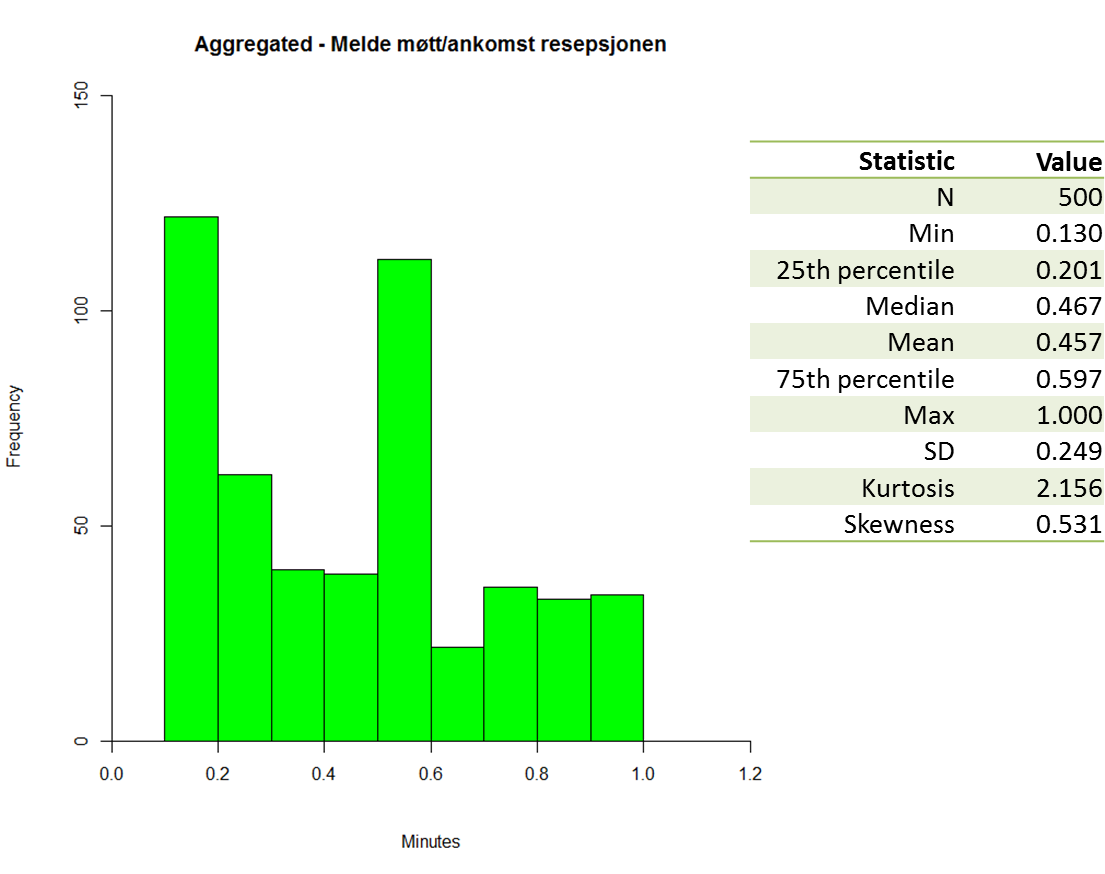


Figure 2 Check in service time distribution (provided by reception staff)


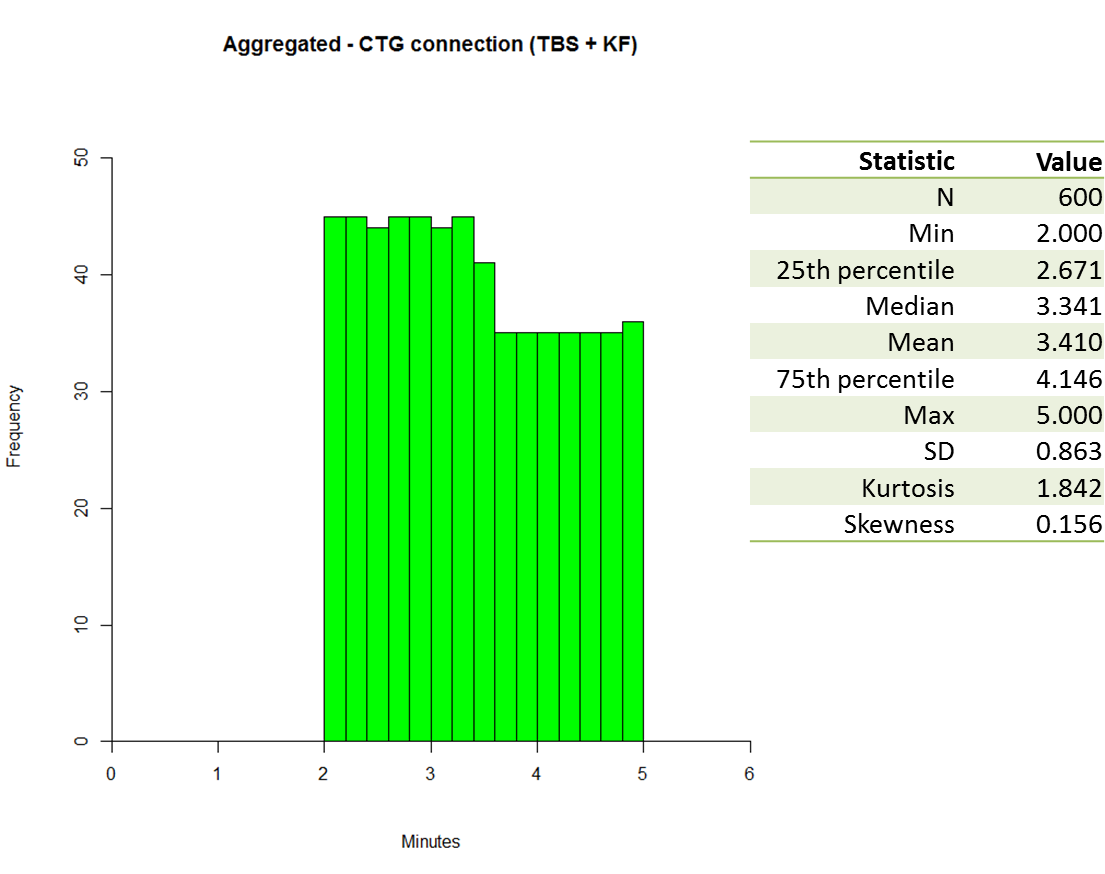


Figure 3 CTG connection service time distribution (provided by TBS and KF)


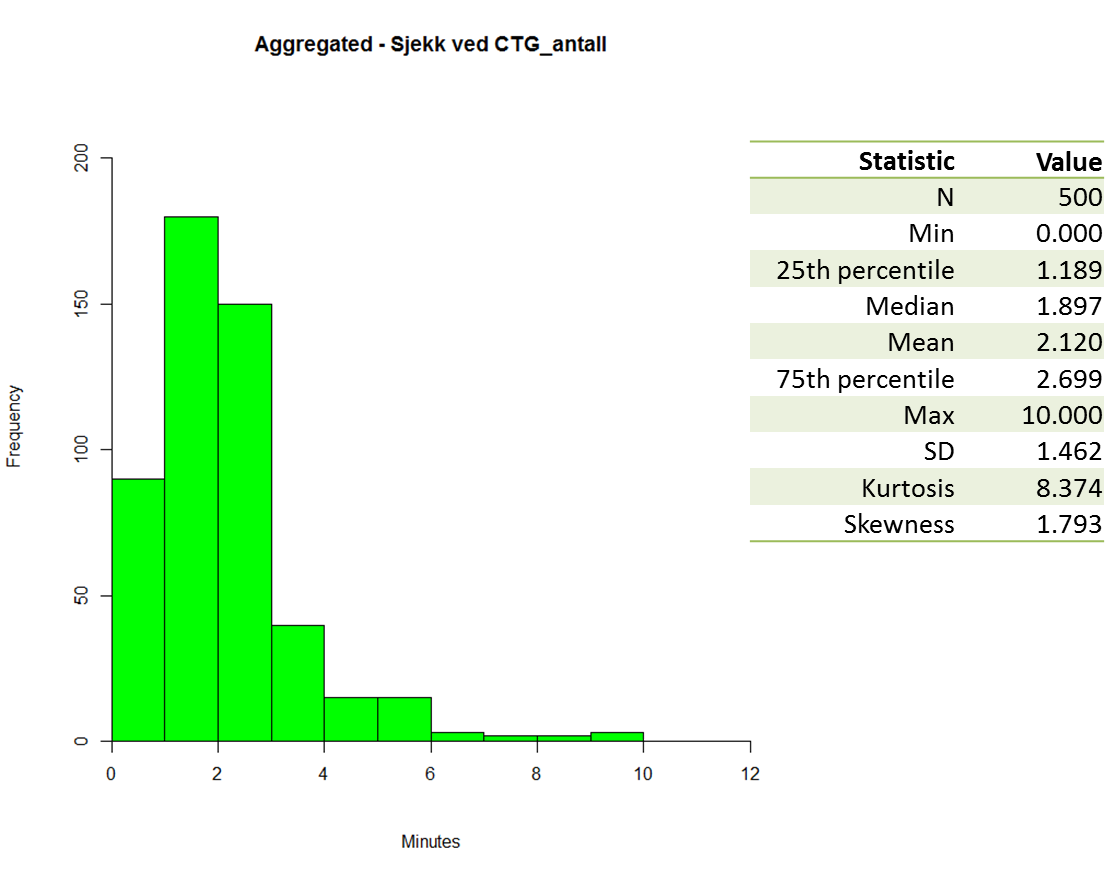


Figure 4 Number of CTG checks (provided by midwives)


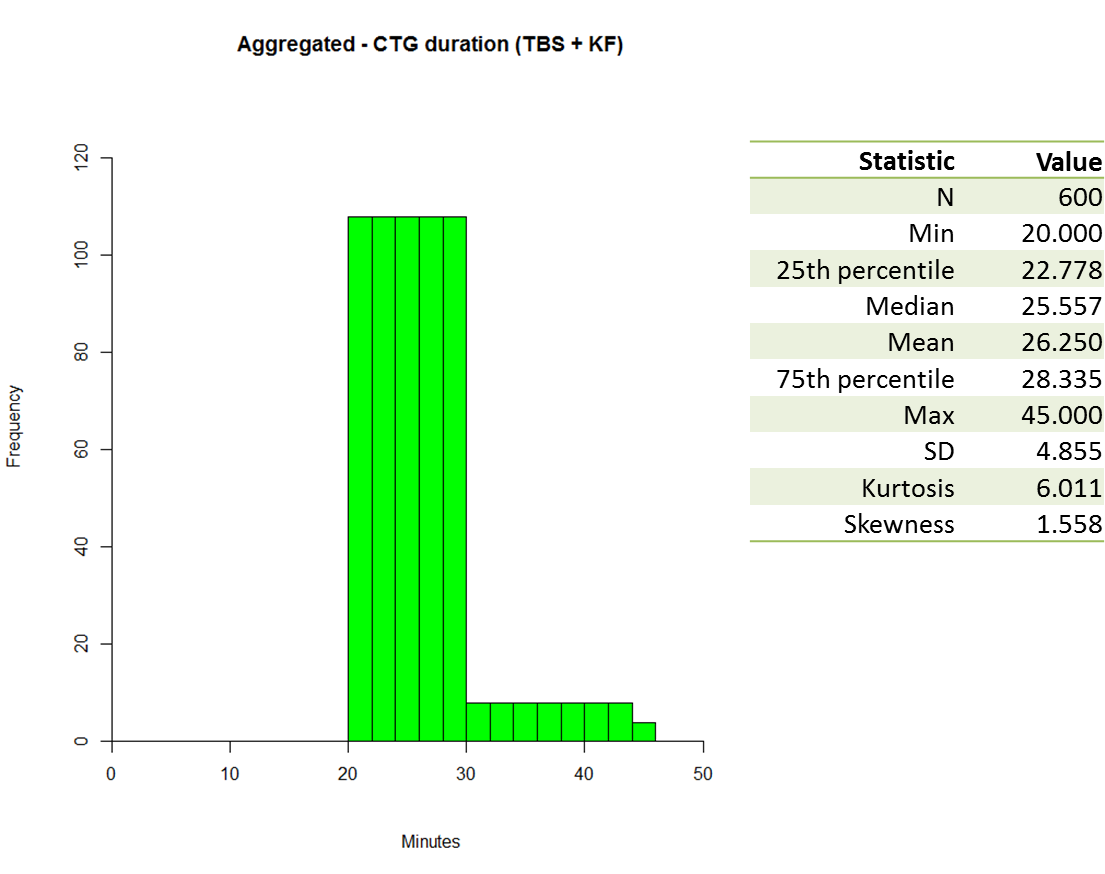


Figure 5 CTG scan duration (provided by TBS and KF)


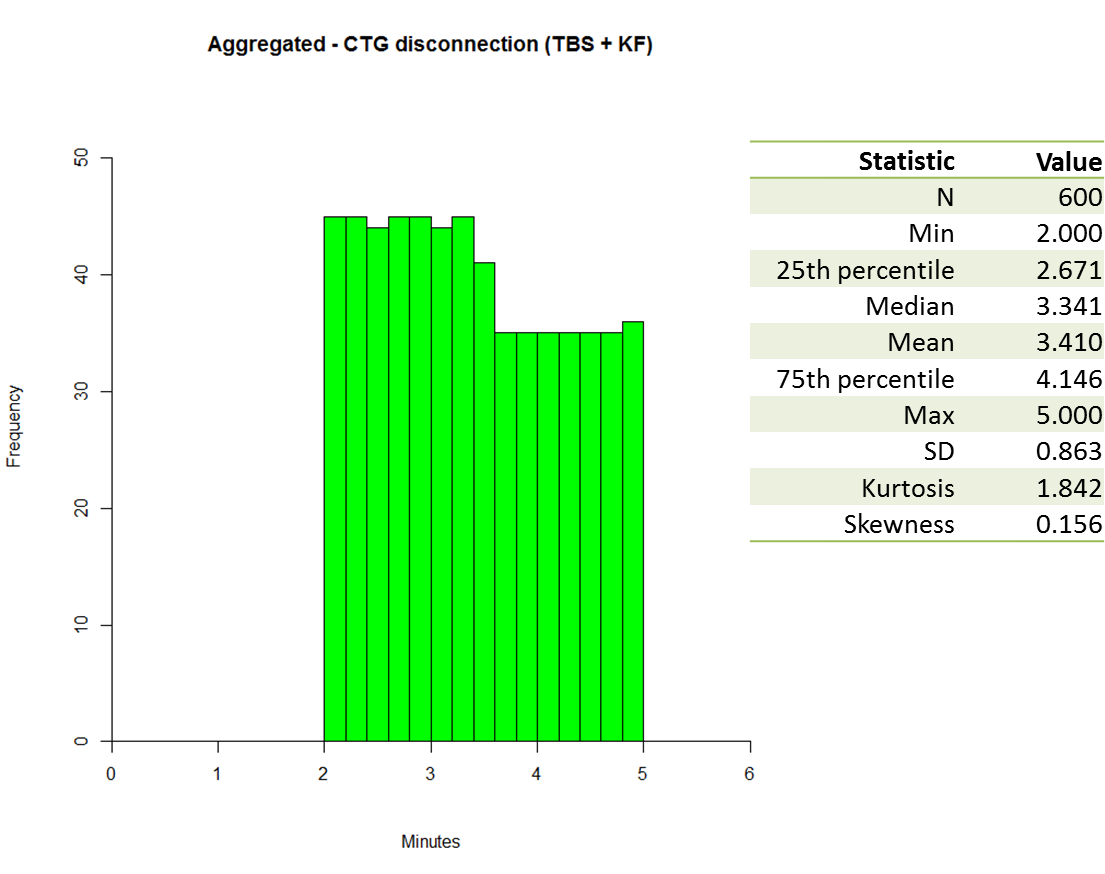


Figure 6 Disconnection from CTG service time distribution (provided by TBS and KF)


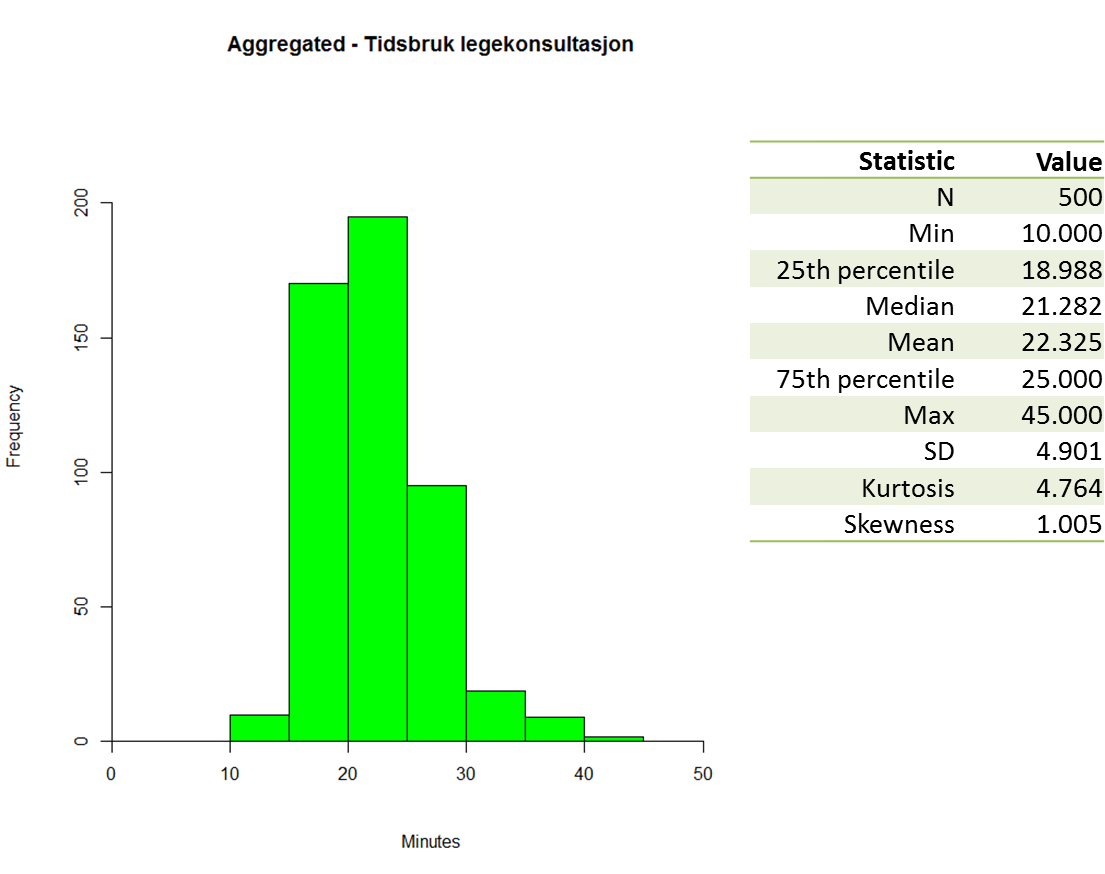


Figure 7 Doctor Consultation service time distribution (provided by doctors)


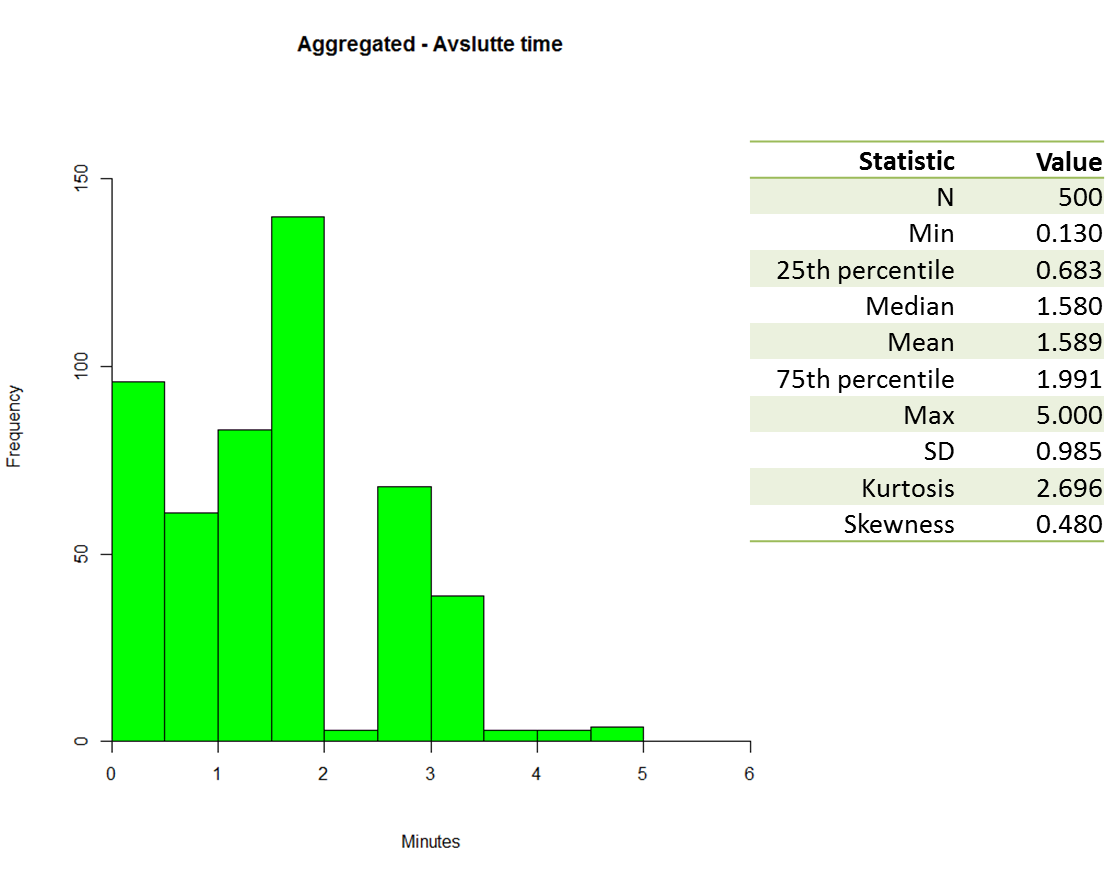


Figure 8 Check out service time distribution (provided by reception staff)


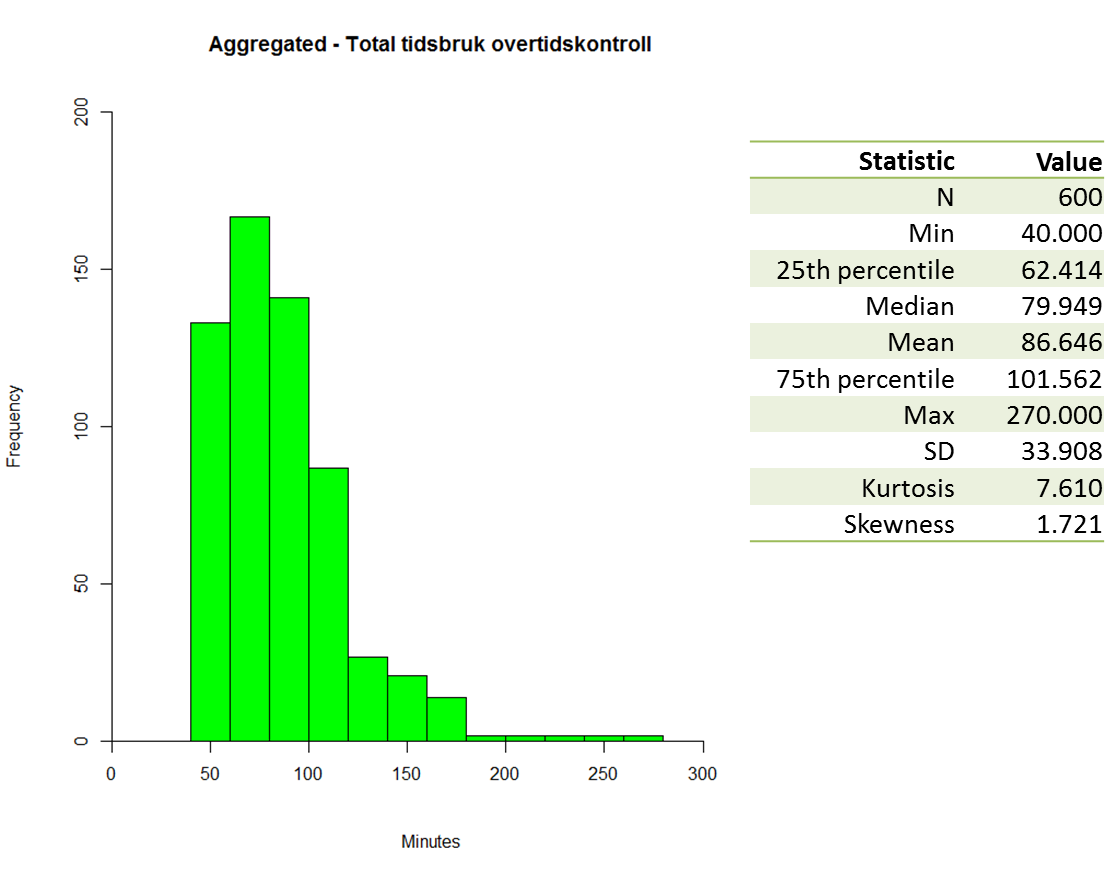


Figure 9 Estimated length of stay (LoS) distribution (provided by midwives)

# Model: Class Structure

The DES clinic model of the Main agent depicted in the UML diagram below is shown in the Model: Graphical overview section.


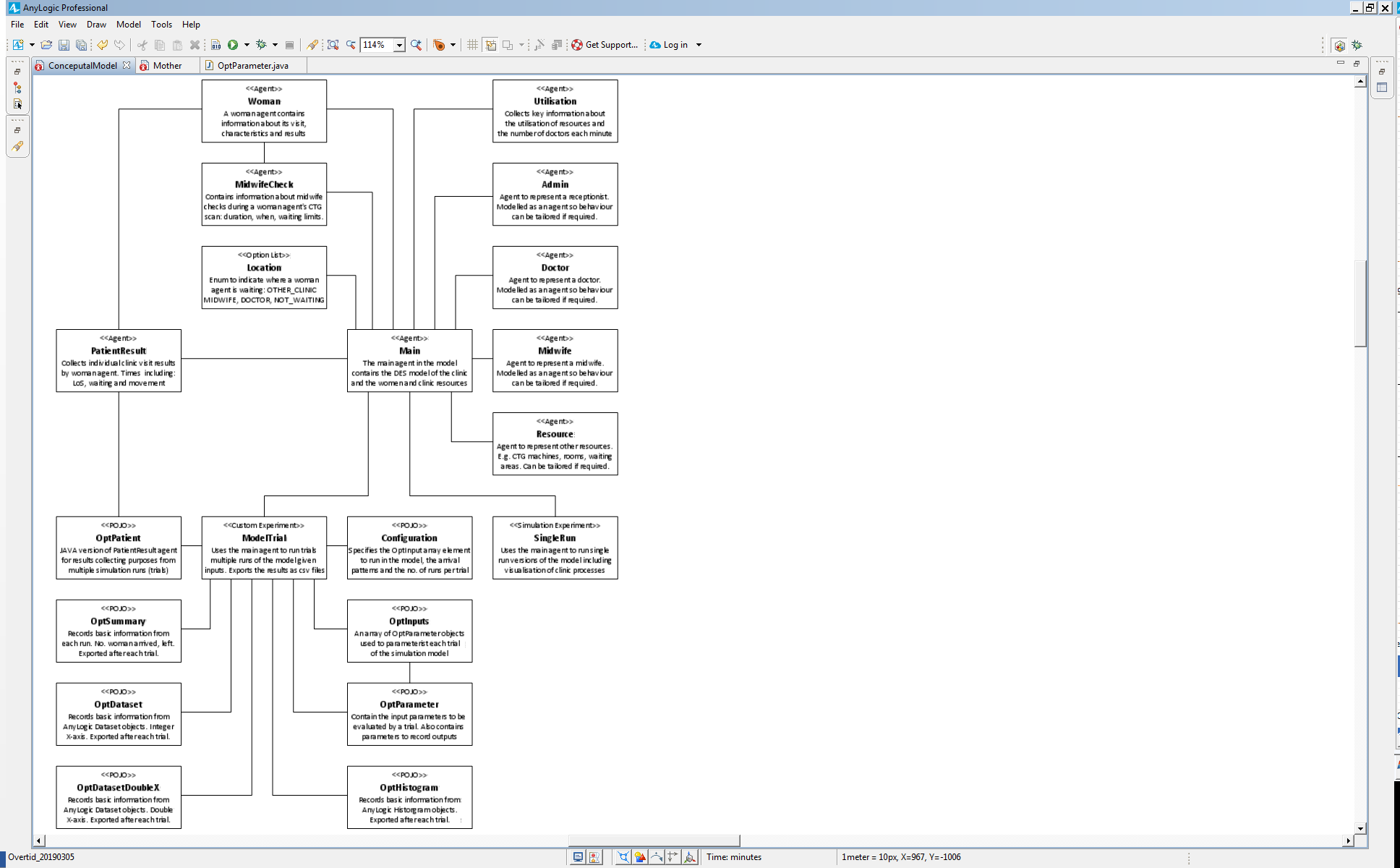


Figure 10 UML type diagram of the post term clinic outpatient model

# Model: Graphical overview

A simplified overview of the DES clinic model is provided below. This diagram omits midwife checks, and does not explain the internal coding of the women agents. This is a more detailed version of Figure 2 included in the paper.


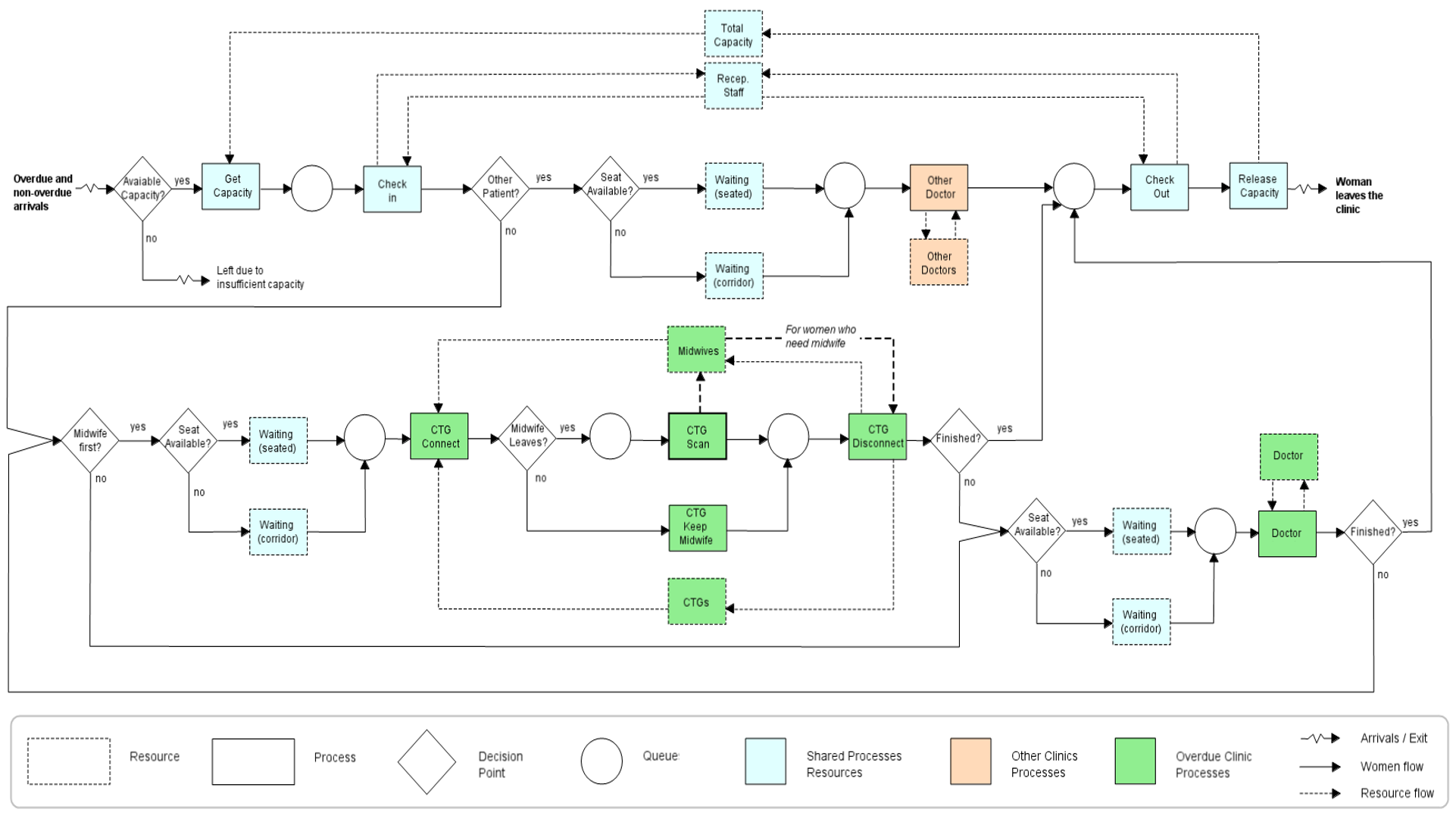


Figure 11 Simplified flow chart of DES clinic model

# Full Factorial experimental design

All the configurations in Table 3 were evaluated in an earlier version of the model. The configurations that are included in the paper and analysed in greater detail are highlighted in green in Table 3, they were renamed in the paper.

Table 1 Configuration inputs

| Var. | Description | Values |
| --- | --- | --- |
| x1 | The women arrival pattern: | 0 = 100% at 08:00  1 = 50% at 08:00 and 50% at 08:30 |
| x2 | The first y patients can see the doctor first.  y = number of doctors, | 0 = no  1 = yes |
| x3 | Number of midwives available: | 2,3,4 |
| x4 | Number of doctors available: | 2,3,4 |
| x5 | Number of CTGs available: | 3,4,5 |

Table 2 Configuration inputs in the model

| Var. | Model variable(s) | Type |
| --- | --- | --- |
| x1 | All_at8 | Boolean: true = 0; false = 1 |
| x2 | doctorBeforeMidwife | Boolean: true = 1; false = 0 |
| x3 | midwives | Int |
| x4 | doc_FØD  doc_FØD2  doc_ØHJ  doc_Extra | Int 0 or 1 |
| x5 | equip_Room026  equip_Roome039 | Int |

Table 3 Configuration highlighted in bold included in the BJOG paper

| No. | x1 | x2 | x3 | x4 | x5 |  | No. | x1 | x2 | x3 | x4 | x5 |
| --- | --- | --- | --- | --- | --- | --- | --- | --- | --- | --- | --- | --- |
| **1** | **0** | **0** | **2** | **2** | **3** |  | **55** | **0** | **1** | **3** | **3** | **4** |
| **2** | **1** | **0** | **2** | **2** | **3** |  | **56** | **1** | **1** | **3** | **3** | **4** |
| **3** | **0** | **1** | **2** | **2** | **3** |  | 57 | 0 | 0 | 4 | 3 | 4 |
| **4** | **1** | **1** | **2** | **2** | **3** |  | 58 | 1 | 0 | 4 | 3 | 4 |
| **5** | **0** | **0** | **3** | **2** | **3** |  | 59 | 0 | 1 | 4 | 3 | 4 |
| **6** | **1** | **0** | **3** | **2** | **3** |  | 60 | 1 | 1 | 4 | 3 | 4 |
| **7** | **0** | **1** | **3** | **2** | **3** |  | 61 | 0 | 0 | 2 | 4 | 4 |
| **8** | **1** | **1** | **3** | **2** | **3** |  | 62 | 1 | 0 | 2 | 4 | 4 |
| 9 | 0 | 0 | 4 | 2 | 3 |  | 63 | 0 | 1 | 2 | 4 | 4 |
| 10 | 1 | 0 | 4 | 2 | 3 |  | 64 | 1 | 1 | 2 | 4 | 4 |
| 11 | 0 | 1 | 4 | 2 | 3 |  | 65 | 0 | 0 | 3 | 4 | 4 |
| 12 | 1 | 1 | 4 | 2 | 3 |  | 66 | 1 | 0 | 3 | 4 | 4 |
| **13** | **0** | **0** | **2** | **3** | **3** |  | 67 | 0 | 1 | 3 | 4 | 4 |
| **14** | **1** | **0** | **2** | **3** | **3** |  | 68 | 1 | 1 | 3 | 4 | 4 |
| **15** | **0** | **1** | **2** | **3** | **3** |  | 69 | 0 | 0 | 4 | 4 | 4 |
| **16** | **1** | **1** | **2** | **3** | **3** |  | 70 | 1 | 0 | 4 | 4 | 4 |
| **17** | **0** | **0** | **3** | **3** | **3** |  | 71 | 0 | 1 | 4 | 4 | 4 |
| **18** | **1** | **0** | **3** | **3** | **3** |  | 72 | 1 | 1 | 4 | 4 | 4 |
| **19** | **0** | **1** | **3** | **3** | **3** |  | 73 | 0 | 0 | 2 | 2 | 5 |
| **20** | **1** | **1** | **3** | **3** | **3** |  | 74 | 1 | 0 | 2 | 2 | 5 |
| 21 | 0 | 0 | 4 | 3 | 3 |  | 75 | 0 | 1 | 2 | 2 | 5 |
| 22 | 1 | 0 | 4 | 3 | 3 |  | 76 | 1 | 1 | 2 | 2 | 5 |
| 23 | 0 | 1 | 4 | 3 | 3 |  | 77 | 0 | 0 | 3 | 2 | 5 |
| 24 | 1 | 1 | 4 | 3 | 3 |  | 78 | 1 | 0 | 3 | 2 | 5 |
| 25 | 0 | 0 | 2 | 4 | 3 |  | 79 | 0 | 1 | 3 | 2 | 5 |
| 26 | 1 | 0 | 2 | 4 | 3 |  | 80 | 1 | 1 | 3 | 2 | 5 |
| 27 | 0 | 1 | 2 | 4 | 3 |  | 81 | 0 | 0 | 4 | 2 | 5 |
| 28 | 1 | 1 | 2 | 4 | 3 |  | 82 | 1 | 0 | 4 | 2 | 5 |
| 29 | 0 | 0 | 3 | 4 | 3 |  | 83 | 0 | 1 | 4 | 2 | 5 |
| 30 | 1 | 0 | 3 | 4 | 3 |  | 84 | 1 | 1 | 4 | 2 | 5 |
| 31 | 0 | 1 | 3 | 4 | 3 |  | 85 | 0 | 0 | 2 | 3 | 5 |
| 32 | 1 | 1 | 3 | 4 | 3 |  | 86 | 1 | 0 | 2 | 3 | 5 |
| 33 | 0 | 0 | 4 | 4 | 3 |  | 87 | 0 | 1 | 2 | 3 | 5 |
| 34 | 1 | 0 | 4 | 4 | 3 |  | 88 | 1 | 1 | 2 | 3 | 5 |
| 35 | 0 | 1 | 4 | 4 | 3 |  | 89 | 0 | 0 | 3 | 3 | 5 |
| 36 | 1 | 1 | 4 | 4 | 3 |  | 90 | 1 | 0 | 3 | 3 | 5 |
| **37** | **0** | **0** | **2** | **2** | **4** |  | 91 | 0 | 1 | 3 | 3 | 5 |
| **38** | **1** | **0** | **2** | **2** | **4** |  | 92 | 1 | 1 | 3 | 3 | 5 |
| **39** | **0** | **1** | **2** | **2** | **4** |  | 93 | 0 | 0 | 4 | 3 | 5 |
| **40** | **1** | **1** | **2** | **2** | **4** |  | 94 | 1 | 0 | 4 | 3 | 5 |
| **41** | **0** | **0** | **3** | **2** | **4** |  | 95 | 0 | 1 | 4 | 3 | 5 |
| **42** | **1** | **0** | **3** | **2** | **4** |  | 96 | 1 | 1 | 4 | 3 | 5 |
| **43** | **0** | **1** | **3** | **2** | **4** |  | 97 | 0 | 0 | 2 | 4 | 5 |
| **44** | **1** | **1** | **3** | **2** | **4** |  | 98 | 1 | 0 | 2 | 4 | 5 |
| 45 | 0 | 0 | 4 | 2 | 4 |  | 99 | 0 | 1 | 2 | 4 | 5 |
| 46 | 1 | 0 | 4 | 2 | 4 |  | 100 | 1 | 1 | 2 | 4 | 5 |
| 47 | 0 | 1 | 4 | 2 | 4 |  | 101 | 0 | 0 | 3 | 4 | 5 |
| 48 | 1 | 1 | 4 | 2 | 4 |  | 102 | 1 | 0 | 3 | 4 | 5 |
| **49** | **0** | **0** | **2** | **3** | **4** |  | 103 | 0 | 1 | 3 | 4 | 5 |
| **50** | **1** | **0** | **2** | **3** | **4** |  | 104 | 1 | 1 | 3 | 4 | 5 |
| **51** | **0** | **1** | **2** | **3** | **4** |  | 105 | 0 | 0 | 4 | 4 | 5 |
| **52** | **1** | **1** | **2** | **3** | **4** |  | 106 | 1 | 0 | 4 | 4 | 5 |
| **53** | **0** | **0** | **3** | **3** | **4** |  | 107 | 0 | 1 | 4 | 4 | 5 |
| **54** | **1** | **0** | **3** | **3** | **4** |  | 108 | 1 | 1 | 4 | 4 | 5 |
