# Supplementary material for: Capacity and patient flow planning in post-term pregnancy outpatient clinics: a computer simulation modelling study
Source: BMC Health Serv Res. 2020 Feb 14;20:117. doi: 10.1186/s12913-020-4943-y (PMC7023739; doi:10.1186/s12913-020-4943-y)
Supplement: Supplementary file 3 — Additional file 3. Mendeley Data; Provides a link to a Mendeley repository where the model, the results and the documentation used in this paper can be found. [file 12913_2020_4943_MOESM3_ESM.docx]

**Additional file 3 – Mendeley Data**

I have submitted the model and associated documents to this dataset. It is currently being SPAM checked.

Viana, Joe; Simonsen, Tone Breines ; Faraas, Hildegunn E.; Schmidt, Nina; Dahl, Fredrik A.; Flo, Kari (2019), “Post-term pregnancy outpatient clinic model”, Mendeley Data, V1, doi: 10.17632/4jc5hz4mp3.1

<https://data.mendeley.com/datasets/4jc5hz4mp3/1>
