# Supplementary material for: Capacity and patient flow planning in post-term pregnancy outpatient clinics: a computer simulation modelling study
Source: BMC Health Serv Res. 2020 Feb 14;20:117. doi: 10.1186/s12913-020-4943-y (PMC7023739; doi:10.1186/s12913-020-4943-y)
Supplement: Supplementary file 4 — Additional file 4. Explanation of Fig. 1 data; Provides an explanation of how Fig. 1 was constructed and summary data. [file 12913_2020_4943_MOESM4_ESM.docx]

**Additional file 4 – Explanation of Figure 1 data**

This document summarises the data used to create Figure 1, shown again below. The data were extracted from two Hospital Information Systems (HIS), DIPS and CSAM Partus between 04/01/2010 to 31/12/2017. The aggregate data used to create the graphs is provided in this document. The raw data cannot be made publically available due to data protection laws.


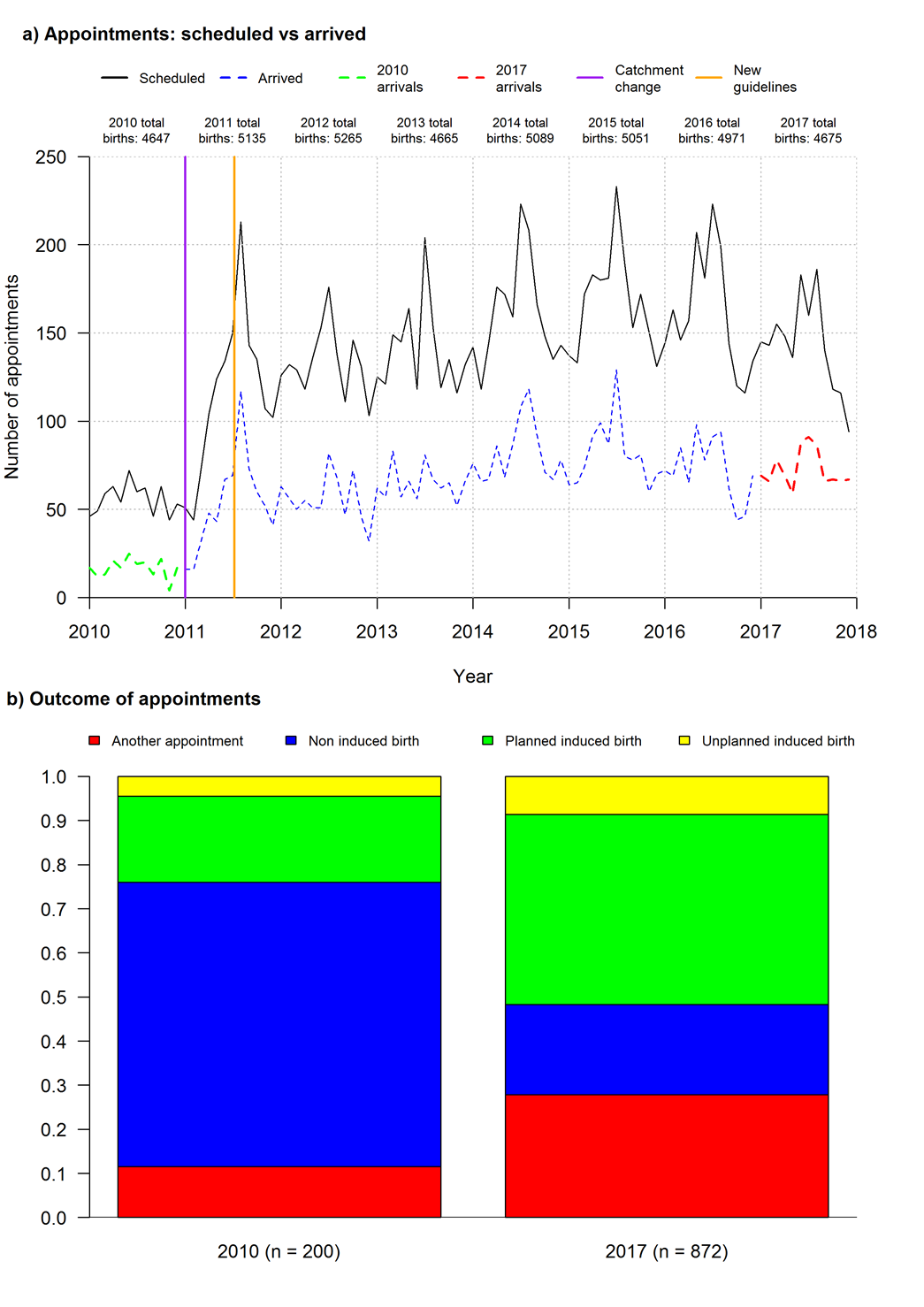


Figure 1: Change in clinic demand and appointment outcomes from 2010 to 2017

Panel a) arrived vs. scheduled appointments, derived from hospital information systems DIPS and CSAM Partus based on ICDM-10-CM Diagnosis Code O48.0.Panel b) appointment outcomes using subsequent O48.0 entries, and procedural codes for induced and non-induced births

**DIPS data**

DIPS is the hospital’s central HIS. The data extract consisted of 24,321 records. Each record contained several variables associated with: a generated ID, dates and times, diagnostic and procedure codes etc. These data relate to 4,809 women, who had an O48 diagnosis, as their main diagnosis or a co-diagnosis. The records in a specified period around a woman’s O48 diagnosis are extracted, to determine the outcome of the appointment (Figure 1b). Some women have more than one pregnancy over the 8 year period and each pregnancy has one or more associated records. Figure 2 summarises how the DIPS data was processed. Table 6b in Figure 2 is the key outcome.


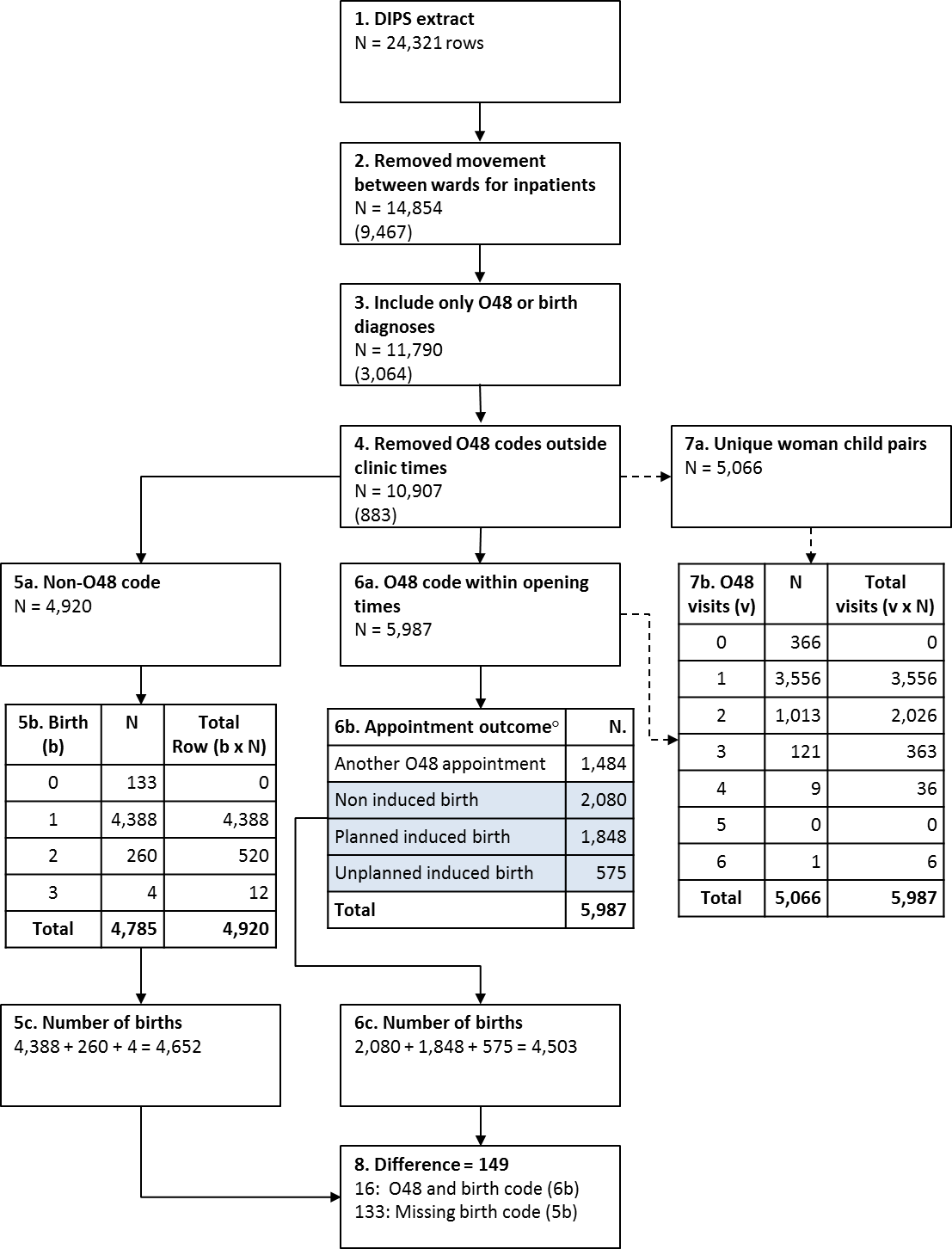


Figure 2: DIPS data, used to create the dashed line (Arrived) in Figure 1a and Figure 1b

**Partus data**

The DIPS data extract captured information about women who attended their O48 appointments and associated delivery records; it did not include information about scheduled appointments that were not attended. To obtain data about scheduled appointments data were extracted from Partus, covering the period 04/01/2010 to 29/12/2017. The Partus extract consisted of 12,970 records. Each record consisted of variables including, a generated ID, main diagnosis, appointment date/times, treatment data/times and categorical outcome of the interaction. The data relate to 9,773 women. The Partus data extract included women with O48 as the main or co-diagnosis. It is therefore assumed that all of the records relate to an O48 woman. The solid line (Scheduled) in Figure 1 is based on the data in box 2 below.


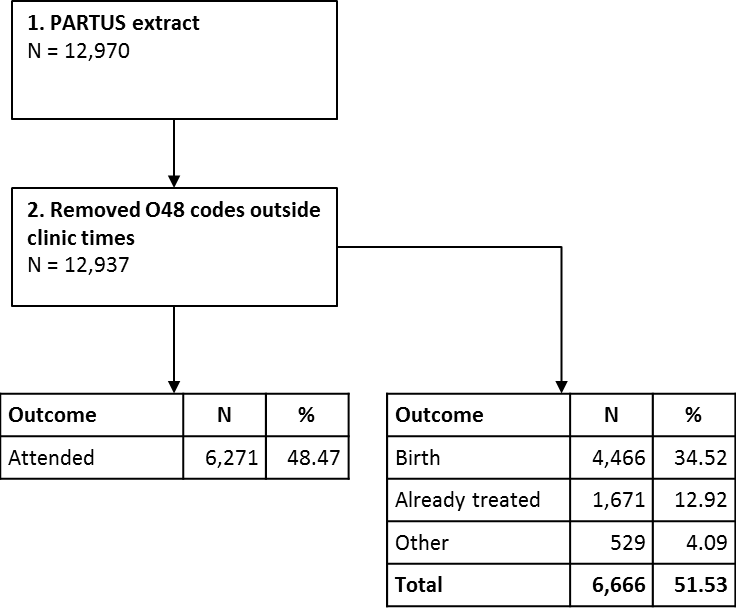


Figure 3: Partus data, used to create the solid line (Scheduled) in Figure 1a

**Arrivals: DIPS vs. Partus data**

The DIPS data were used to analyse the outcome of each visit as the extract contained the appropriate data, all visits/episode. Figure 4 compares the arrivals identified in the DIPS and Partus data. The DIPS data were chosen to indicate the arrivals in Figure 1a as the outcomes in Figure 1b were derived from this data. The total number and pattern was deemed close enough for the purpose of Figure 1.


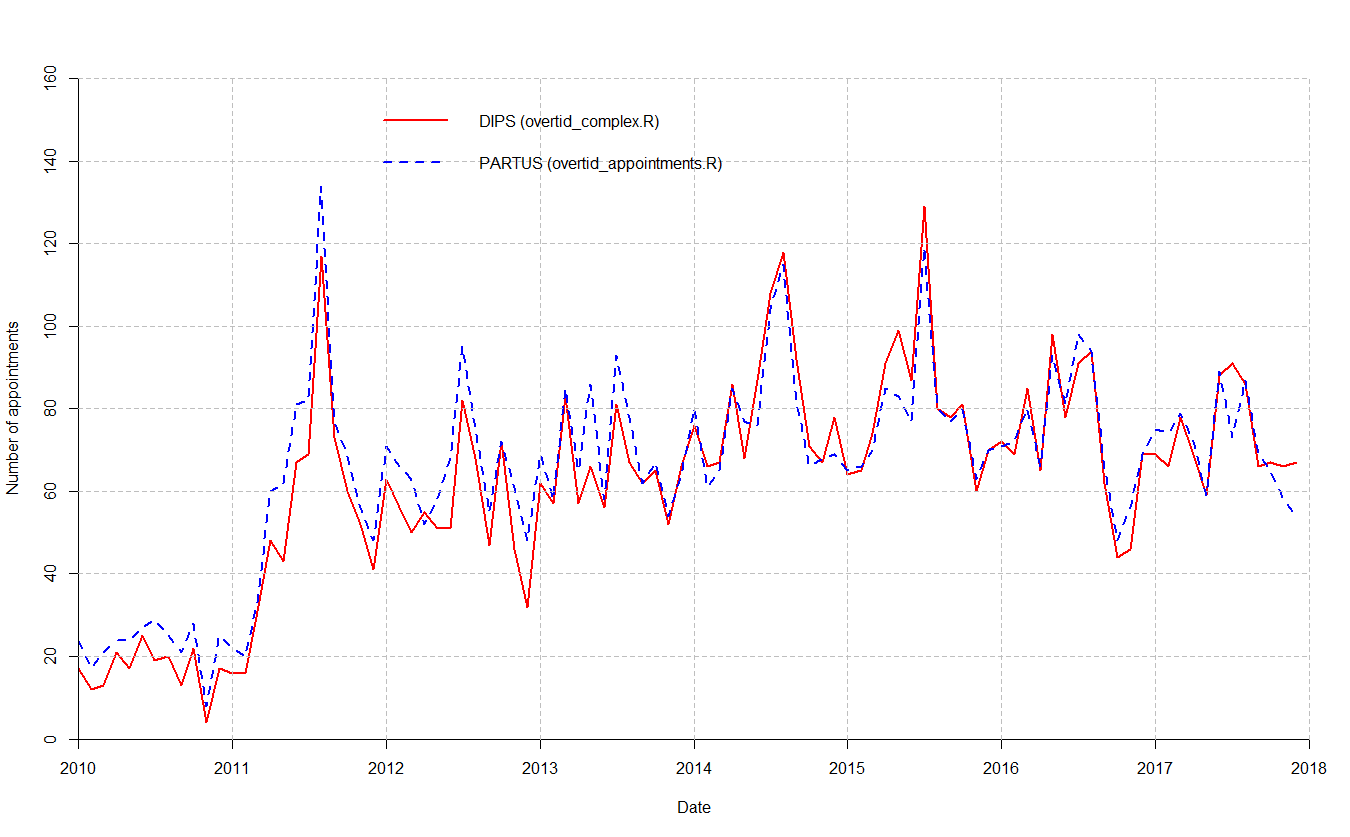


Figure 4: Comparison between the DIPS and Partus arrival data

**Raw Data**

The raw data from DIPS and Partus are not available due to data protection law. The summary information derived from the raw data used to create Figure 1 is shown in Table 1 and Table 2.

Table 1: DIPS data used to create Figure 1b

| **Year** | **Another O48**  **appointment** | **Non-Induced**  **Birth** | **Planned Induced**  **Birth** | **Unplanned Induced**  **Birth** |
| --- | --- | --- | --- | --- |
| 2010 | 0.115 | 0.645 | 0.195 | 0.045 |
| 2017 | 0.279 | 0.204 | 0.431 | 0.086 |

Table 2: Partus data (scheduled) and DIPs data (arrived) used to create Figure 1a.

| **year** | **month** | **scheduled** | **arrived** | **x_label** | **x** |
| --- | --- | --- | --- | --- | --- |
| 2010 | 1 | 46 | 17 | 01.01.2010 | 1 |
| 2010 | 2 | 49 | 12 | 01.02.2010 | 2 |
| 2010 | 3 | 59 | 13 | 01.03.2010 | 3 |
| 2010 | 4 | 63 | 21 | 01.04.2010 | 4 |
| 2010 | 5 | 54 | 17 | 01.05.2010 | 5 |
| 2010 | 6 | 72 | 25 | 01.06.2010 | 6 |
| 2010 | 7 | 60 | 19 | 01.07.2010 | 7 |
| 2010 | 8 | 62 | 20 | 01.08.2010 | 8 |
| 2010 | 9 | 46 | 13 | 01.09.2010 | 9 |
| 2010 | 10 | 63 | 22 | 01.10.2010 | 10 |
| 2010 | 11 | 44 | 4 | 01.11.2010 | 11 |
| 2010 | 12 | 53 | 17 | 01.12.2010 | 12 |
| 2011 | 1 | 51 | 16 | 01.01.2011 | 13 |
| 2011 | 2 | 44 | 16 | 01.02.2011 | 14 |
| 2011 | 3 | 71 | 31 | 01.03.2011 | 15 |
| 2011 | 4 | 104 | 48 | 01.04.2011 | 16 |
| 2011 | 5 | 124 | 43 | 01.05.2011 | 17 |
| 2011 | 6 | 134 | 67 | 01.06.2011 | 18 |
| 2011 | 7 | 150 | 69 | 01.07.2011 | 19 |
| 2011 | 8 | 213 | 117 | 01.08.2011 | 20 |
| 2011 | 9 | 143 | 73 | 01.09.2011 | 21 |
| 2011 | 10 | 135 | 60 | 01.10.2011 | 22 |
| 2011 | 11 | 107 | 52 | 01.11.2011 | 23 |
| 2011 | 12 | 102 | 41 | 01.12.2011 | 24 |
| 2012 | 1 | 126 | 63 | 01.01.2012 | 25 |
| 2012 | 2 | 132 | 56 | 01.02.2012 | 26 |
| 2012 | 3 | 129 | 50 | 01.03.2012 | 27 |
| 2012 | 4 | 118 | 55 | 01.04.2012 | 28 |
| 2012 | 5 | 136 | 51 | 01.05.2012 | 29 |
| 2012 | 6 | 153 | 51 | 01.06.2012 | 30 |
| 2012 | 7 | 176 | 82 | 01.07.2012 | 31 |
| 2012 | 8 | 138 | 68 | 01.08.2012 | 32 |
| 2012 | 9 | 111 | 47 | 01.09.2012 | 33 |
| 2012 | 10 | 146 | 72 | 01.10.2012 | 34 |
| 2012 | 11 | 131 | 46 | 01.11.2012 | 35 |
| 2012 | 12 | 103 | 32 | 01.12.2012 | 36 |
| 2013 | 1 | 125 | 62 | 01.01.2013 | 37 |
| 2013 | 2 | 121 | 57 | 01.02.2013 | 38 |
| 2013 | 3 | 149 | 83 | 01.03.2013 | 39 |
| 2013 | 4 | 145 | 57 | 01.04.2013 | 40 |
| 2013 | 5 | 164 | 66 | 01.05.2013 | 41 |
| 2013 | 6 | 118 | 56 | 01.06.2013 | 42 |
| 2013 | 7 | 204 | 81 | 01.07.2013 | 43 |
| 2013 | 8 | 153 | 67 | 01.08.2013 | 44 |
| 2013 | 9 | 119 | 62 | 01.09.2013 | 45 |
| 2013 | 10 | 135 | 65 | 01.10.2013 | 46 |
| 2013 | 11 | 116 | 52 | 01.11.2013 | 47 |
| 2013 | 12 | 132 | 66 | 01.12.2013 | 48 |
| 2014 | 1 | 142 | 76 | 01.01.2014 | 49 |
| 2014 | 2 | 118 | 66 | 01.02.2014 | 50 |
| 2014 | 3 | 144 | 67 | 01.03.2014 | 51 |
| 2014 | 4 | 176 | 86 | 01.04.2014 | 52 |
| 2014 | 5 | 172 | 68 | 01.05.2014 | 53 |
| 2014 | 6 | 159 | 87 | 01.06.2014 | 54 |
| 2014 | 7 | 223 | 108 | 01.07.2014 | 55 |
| 2014 | 8 | 208 | 118 | 01.08.2014 | 56 |
| 2014 | 9 | 166 | 92 | 01.09.2014 | 57 |
| 2014 | 10 | 148 | 71 | 01.10.2014 | 58 |
| 2014 | 11 | 135 | 67 | 01.11.2014 | 59 |
| 2014 | 12 | 143 | 78 | 01.12.2014 | 60 |
| 2015 | 1 | 137 | 64 | 01.01.2015 | 61 |
| 2015 | 2 | 133 | 65 | 01.02.2015 | 62 |
| 2015 | 3 | 172 | 74 | 01.03.2015 | 63 |
| 2015 | 4 | 183 | 91 | 01.04.2015 | 64 |
| 2015 | 5 | 180 | 99 | 01.05.2015 | 65 |
| 2015 | 6 | 181 | 87 | 01.06.2015 | 66 |
| 2015 | 7 | 233 | 129 | 01.07.2015 | 67 |
| 2015 | 8 | 190 | 80 | 01.08.2015 | 68 |
| 2015 | 9 | 153 | 78 | 01.09.2015 | 69 |
| 2015 | 10 | 172 | 81 | 01.10.2015 | 70 |
| 2015 | 11 | 151 | 60 | 01.11.2015 | 71 |
| 2015 | 12 | 131 | 70 | 01.12.2015 | 72 |
| 2016 | 1 | 144 | 72 | 01.01.2016 | 73 |
| 2016 | 2 | 163 | 69 | 01.02.2016 | 74 |
| 2016 | 3 | 146 | 85 | 01.03.2016 | 75 |
| 2016 | 4 | 157 | 65 | 01.04.2016 | 76 |
| 2016 | 5 | 207 | 98 | 01.05.2016 | 77 |
| 2016 | 6 | 181 | 78 | 01.06.2016 | 78 |
| 2016 | 7 | 223 | 91 | 01.07.2016 | 79 |
| 2016 | 8 | 199 | 94 | 01.08.2016 | 80 |
| 2016 | 9 | 144 | 62 | 01.09.2016 | 81 |
| 2016 | 10 | 120 | 44 | 01.10.2016 | 82 |
| 2016 | 11 | 116 | 46 | 01.11.2016 | 83 |
| 2016 | 12 | 134 | 69 | 01.12.2016 | 84 |
| 2017 | 1 | 145 | 69 | 01.01.2017 | 85 |
| 2017 | 2 | 143 | 66 | 01.02.2017 | 86 |
| 2017 | 3 | 155 | 78 | 01.03.2017 | 87 |
| 2017 | 4 | 148 | 69 | 01.04.2017 | 88 |
| 2017 | 5 | 136 | 59 | 01.05.2017 | 89 |
| 2017 | 6 | 183 | 88 | 01.06.2017 | 90 |
| 2017 | 7 | 160 | 91 | 01.07.2017 | 91 |
| 2017 | 8 | 186 | 86 | 01.08.2017 | 92 |
| 2017 | 9 | 140 | 66 | 01.09.2017 | 93 |
| 2017 | 10 | 118 | 67 | 01.10.2017 | 94 |
| 2017 | 11 | 116 | 66 | 01.11.2017 | 95 |
| 2017 | 12 | 94 | 67 | 01.12.2017 | 96 |
